# How Doping Regulates As(III) Adsorption at TiO_2_ Surfaces: A DFT + U Study

**DOI:** 10.3390/molecules29173991

**Published:** 2024-08-23

**Authors:** Xiaoxiao Huang, Mengru Wu, Rongying Huang, Gang Yang

**Affiliations:** College of Resources and Environment, Southwest University, Chongqing 400715, China; huangxx95105@163.com (X.H.); hry17265669392@163.com (R.H.)

**Keywords:** arsenic pollution, TiO_2_, adsorption, doping, crystal control

## Abstract

The efficient adsorption and removal of As(III), which is highly toxic, remains difficult. TiO_2_ shows promise in this field, though the process needs improvement. Herein, how doping regulates As(OH)_3_ adsorption over TiO_2_ surfaces is comprehensively investigated by means of the DFT + D3 approach. Doping creates the bidentate mononuclear (Ce doping at the Ti_5c_ site), tridentate (N, S doping at the O_2c_ site), and other new adsorption structures. The extent of structural perturbation correlates with the atomic radius when doping the Ti site (Ce >> Fe, Mn, V >> B), while it correlates with the likelihood of forming more bonds when doping the O site (N > S > F). Doping the O_2c_, O_3c_ rather than the Ti_5c_ site is more effective in enhancing As(OH)_3_ adsorption and also causes more structural perturbation and diversity. Similar to the scenario of pristine surfaces, the bidentate binuclear complexes with two Ti-O_As_ bonds are often the most preferred, except for B doping at the Ti_5c_ site, S doping at the O_2c_ site, and B doping at the O_3c_ site of rutile (110) and Ce, B doping at the Ti_5c_ site, N, S doping at the O_2c_ site, and N, S, B doping at the O_3c_ site of anatase (101). Doping significantly regulates the As(OH)_3_ adsorption efficacy, and the adsorption energies reach −4.17, −4.13, and −4.67 eV for Mn doping at the Ti_5c_ site and N doping at the O_2c_ and O_3c_ sites of rutile (110) and −1.99, −2.29, and −2.24 eV for Ce doping at the Ti_5c_ site and N doping at the O_2c_ and O_3c_ sites of anatase (101), respectively. As(OH)_3_ adsorption and removal are crystal-dependent and become apparently more efficient for rutile vs. anatase, whether doped at the Ti_5c_, O_2c_, or O_3c_ site. The auto-oxidation of As(III) occurs when the As centers interact directly with the TiO_2_ surface, and this occurs more frequently for rutile rather than anatase. The multidentate adsorption of As(OH)_3_ causes electron back-donation and As(V) re-reduction to As(IV). The regulatory effects of doping during As(III) adsorption and the critical roles played by crystal control are further unraveled at the molecular level. Significant insights are provided for As(III) pollution management via the adsorption and rational design of efficient scavengers.

## 1. Introduction

Arsenic (As) is a ubiquitously distributed toxic metalloid. Its pollution has seriously endangered the ecological environment and human health and aroused global concern [1,2]. In order to remediate As-polluted sites, a number of techniques have been developed, such as adsorption, precipitation, flocculation, membrane separation, and reverse osmosis [3,4,5]. Among them, adsorption by minerals pronouncedly regulates arsenic migration, bioavailability, and fate and ranks to be the most widely used technique for pollution management [6,7,8]. Inorganic arsenic in natural environments predominates in the As(III) and As(V) forms. Although much more toxic, As(III) is highly mobile and more difficult for adsorption and removal because it exists predominantly as the uncharged As(OH)_3_ moiety [9,10,11]. It thus represents an imperative task to explore the usage efficient As(III) scavengers.

**Figure 1:**
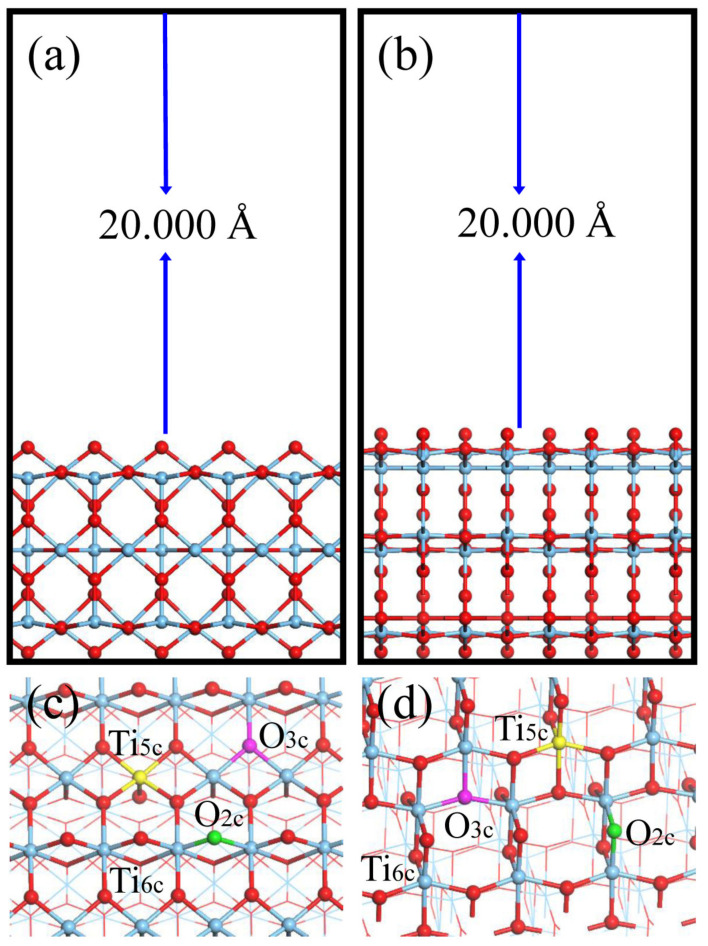
Periodic models for (**a**) rutile (110) and (**b**) anatase (101) surfaces, as well as corresponding top-surface structures. Color scheme: Ti (blue) and O (red). In (**c**,**d**), different types of surface atoms are marked: O_2c_ (two-fold O atom, highlighted in green); O_3c_ (three-fold O atom, highlighted in purple); Ti_5c_ (five-fold Ti atom, highlighted in yellow); and Ti_6c_ (six-fold Ti atom, highlighted in blue).

Metal (hydr)oxides [12,13], zero-valent iron [14,15], zeolites [7,16], clay minerals [17,18], and activated carbon [19,20] have been used for As(III) adsorption. However, they generally suffer from limited adsorption capacity and strength, and hence chemical modification is necessitated [21,22,23]. Mn doping of γ-Al_2_O_3_ enables the transfer of more electrons into a stable status, which further promotes As(III) adsorption [24]. As indicated by periodic density functional theory (DFT) calculations, doping of gibbsite results in strong M-3*d* and O_As_-2*p* orbital interactions (M = Fe(III), Mn(III), Mn(IV)) that facilitate As(III) adsorption [25]. Owing to its superior stability, non-toxicity, and high activity, titanium dioxide (TiO_2_) has been applied extensively for environment-associated adsorption and catalysis [26,27,28,29,30]. Pena et al. [27] found that 0.5 mmol/g of As(III) is adsorbed by nanocrystalline TiO_2_, and the adsorption thermodynamics and kinetics can be well described by the Freundlich isotherm and pseudo-second-order model. Wu et al. [28] showed that due to adsorption by TiO_2_ nanoparticles, the accumulation of As in plants reduces by 40~90%. The controllable configuration of TiO_2_ nanoparticles was realized using 3D-printing technology, and after being used more than 10 times, the TiO_2_ nanoparticles remained effective for the treatment of raw-arsenic-polluted groundwater samples [29]. X-ray absorption spectra (XAS) revealed that As(III) at the TiO_2_ surface tends to adopt the bidentate binuclear configuration, and the Ti-As distance is approximately 3.32 Å [30], which is in good agreement with DFT calculated results [31,32,33]. A density of states (DOS) analysis [34] demonstrated that As(III) at the TiO_2_ surface generates anti-bonding orbitals above the Fermi level, and the Ti-O_As_ bonds are attributed mainly to the electron sharing between the O-*2p* and Ti-*3d* orbitals. Doping is widely documented to be capable of regulating the adsorption and catalytic performances of TiO_2_, e.g., Fe [35,36], Ce [37], B [38], V [39], and Mn [39] at the Ti site and N [40,41], F [42], and S [43] at the O site. Owing to the high efficiency of H_2_O_2_ utilization (99.1%), almost all As(III) adsorbed at the Ce-doped TiO_2_ surface is catalytically oxidized to As(V), and the activity of Ce-doped TiO_2_ remains after five cycles [37]. The incorporation of Fe into the TiO_2_ lattice improves the adsorption capacity of As by arresting the grain growth, which results in a higher affinity [44]. N doping into the TiO_2_ lattice effectively promotes the decolorization of double azo reactive black 5 (RB5) dye and shows significant bactericidal activity against Escherichia coli, with an inhibition rate of up to 92.47% [45]. F doping of TiO_2_ significantly enhances the adsorption capacity of Pb(II) [42], while S-doped TiO_2_ causes a redshift of the optical absorption edge, and hepatotoxin microcystin-LR is efficiently degraded under visible-light irradiation [43]. Despite their wide application, how these dopants within TiO_2_ affect As(III) adsorption and the associated structure–activity relationship remain enigmatic. DFT approaches have been testified to be well suited for structural engineering, mechanistic unraveling, and catalyst design due to their atomic-scale spatial resolution and absolute accuracy (<4.0 kJ/mol) [46,47]. In this study, DFT calculations were carried out, considering (1) two dominant polymorphs of TiO_2_ (rutile and anatase) to probe crystal dependence during As(III) adsorption; (2) all types of doping (at the Ti_5c_, O_2c_, and O_3c_ sites, see Figure 1), to explore the most effective type of doping and site specificity for As(III) adsorption; and (3) a number of dopants, including those aforementioned, to establish the structure–activity relationship during As(III) adsorption. Then, the regulatory mechanisms of doping and crystal control during As(III) adsorption were unraveled at the molecular level. The results provide valuable insight into As(III) adsorption and removal by TiO_2_-based materials and feed back for the design of efficient scavengers for As(III) and other pollutants.

## 2. Results and Discussion

### 2.1. As(OH)_3_ Adsorption by Rutile (110) with the Ti Site Being Doped

**Figure 2:**
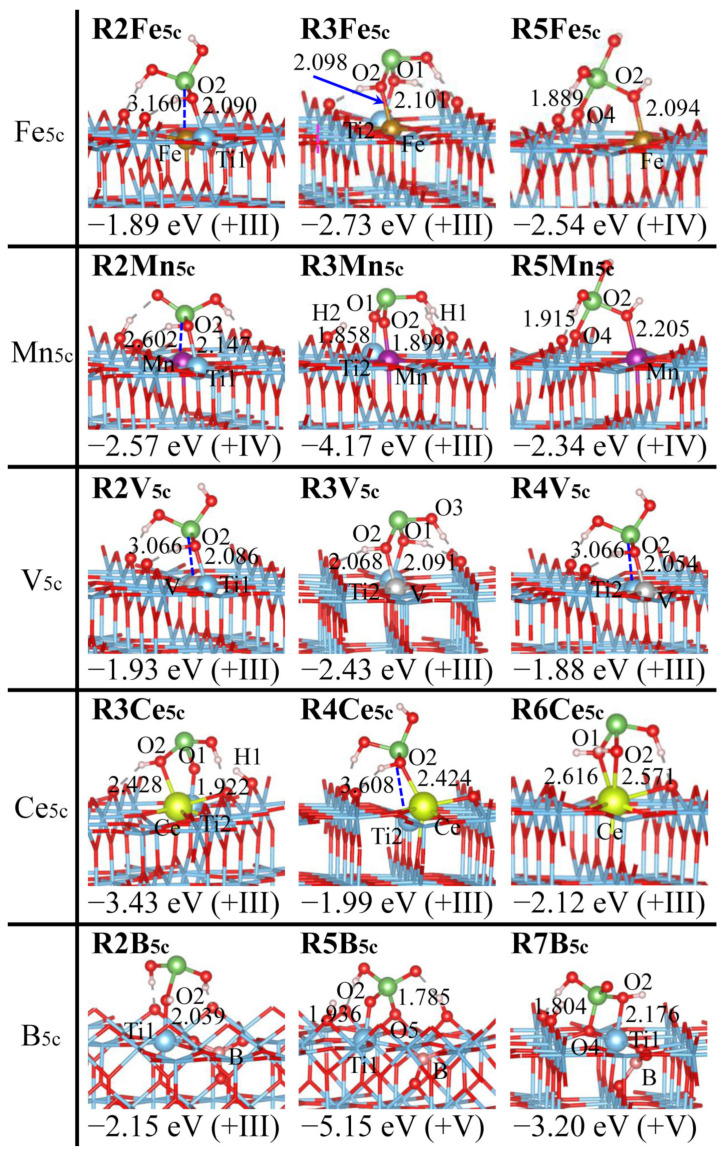
Adsorption configurations of As(OH)_3_ over rutile (110) surface with Ti_5c_ site being doped (D_Ti_ = Fe, Mn, V, Ce, B), together with adsorption energies (*E*_ad_) and oxidation states for the As centers (in parentheses). Color scheme: Ti (blue), O (red), As (green), H (white), Fe (golden), Mn (purple), V (silvery), Ce (yellow), and B (pink). Selected H-bonds are indicated by dashed gray lines. Distances are given in Å.

Adsorption configurations of As(OH)_3_ at the rutile (110) surface with the Ti_5c_ site being doped (D_Ti_ = Fe, Mn, V, Ce, B) are depicted in Figure 2 and Figure S2. They exhibit a marked difference from those of a pristine surface (Figure S3), wherein **R1Pr**, **R2Pr**, and **R3Pr** correspond to the physisorbed, monodentate, and bidentate binuclear complexes [31].

For Fe_5c_ doping, **R2Fe_5c_** with the Ti1-O2 bond remains structurally similar as it appears at a pristine surface (**R2Pr**), while the other adsorption structures may be distinct: **R1Fe_5c_** with the Fe-As interaction vanishes, **R4Fe_5c_** with the Fe-O2 bond becomes monodentate, and **R3Fe_5c_** (Ti2-O1 + Fe-O2) and **R5Fe_5c_** (Fe-O2 + As-O4) are bidentate binuclear. **R2Fe_5c_** and **R4Fe_5c_** are further stabilized by the Fe-As (3.160 Å) and Ti2-As (3.047 Å) interactions, and **R5Fe_5c_** is featured by the As-O_S_ bond that is absent at a pristine surface (O_S_ refers to the surface O atom). Mn_5c_ and V_5c_ doping has As(OH)_3_ adsorption that resembles Fe_5c_ doping, while Ce_5c_ and B_5c_ doping differs substantially, which originates from the divergence of their atomic radii: Ce (1.82 Å) >> Ti and V, Mn, Fe (1.17~1.32 Å) >> B (0.82 Å). Owing to the large atomic radius, Ce protrudes outside the plane of its bonded O_S_ atoms (Figure S4) [48], and the interaction with As(OH)_3_ is promoted so that **R6Ce_5c_** of the bidentate mononuclear motif is generated. However, **R6Ce_5c_** is disfavored sterically by the hepta-coordination for Ce, and this can be reflected by the longer Ce-O_As_ bonds and a less negative *E*_ad_ than in **R3Ce_5c_** of the bidentate binuclear motif (2.616, 2.571 vs. 2.428 Å, and −2.12 vs. −3.43 eV). B has the smallest atomic radius and forms only three B-O_S_ bonds [49], which affects the coordination environment and reactivity of the adjacent surface atoms (Figure S5). B is not directly involved during adsorption, and hence **R3B_5c_** and **R4B_5c_** vanish while the B-As interaction lacks in **R2B_5c_**. **R7B_5c_** arises due to O4 transformation from O_3c_ to O_2c_, and albeit with similar bonding interactions as **R5B_5c_**, it is less preferred due to a four-membered ring (Ti1O2AsO4): *E*_ad_ = −3.20 vs. −5.15 eV.

The stability of different As(OH)_3_ adsorption configurations generally declines as bidentate (**R3**, **R5**, **R6**, **R7**) > monodentate (**R2**, **R4**) > physisorbed (**R1**). **R3** of the bidentate binuclear motif is the most favorable for all dopants at a pristine surface (Figure 2 and Figure S2), which is consistent with EXAFS and ATR-FTIR observations [30,50,51]. The *E*_ad_ amounts to −2.43, −2.73, −3.43, −3.79, and −4.17 eV for **R3V_5c_**, **R3Fe_5c_**, **R3Ce_5c_**, **R3Pr**, and **R3Mn_5c_**, respectively [50]. This agrees with the exergonic nature of As(III) adsorption onto Fe-doped TiO_2_(B) [52]. As(OH)_3_ adsorption in **R3Mn_5c_** is further enhanced by the auto-transfer of H1 and H2 to the O_S_ atoms [31]. Hence, the adsorption efficacy of As(OH)_3_ is regulated pronouncedly by doping the Ti_5c_ site and varies within a wide range. B_5c_ doping is an exception, and **R5B_5c_** formation is strongly benefited: the reactivity of the Ti1 site is enhanced due to the elongation of Ti1-O_S_ bonds (Figure S5) and the superior stability of the AsO_4_ tetrahedron. The O2AsO5 angle in **R5B_5c_** is closer to 109.5° for the regular tetrahedron than in the other adsorption configurations: 103.7° vs. 93.8°, 94.4°, 93.7°, 79.7° for **R5B_5c_** vs. **R5Fe_5c_**, **R5Mn_5c_**, **R5V_5c_**, and **R5Ce_5c_**. The deviation degree of the O2AsO5 angle also interprets the lowest relative stability of **R5** vs. **R3** for Ce_5c_ doping (Δ*E*_ad_ = 1.49 eV).

### 2.2. As(OH)_3_ Adsorption by Rutile (110) with Doping the O Site

**Figure 3:**
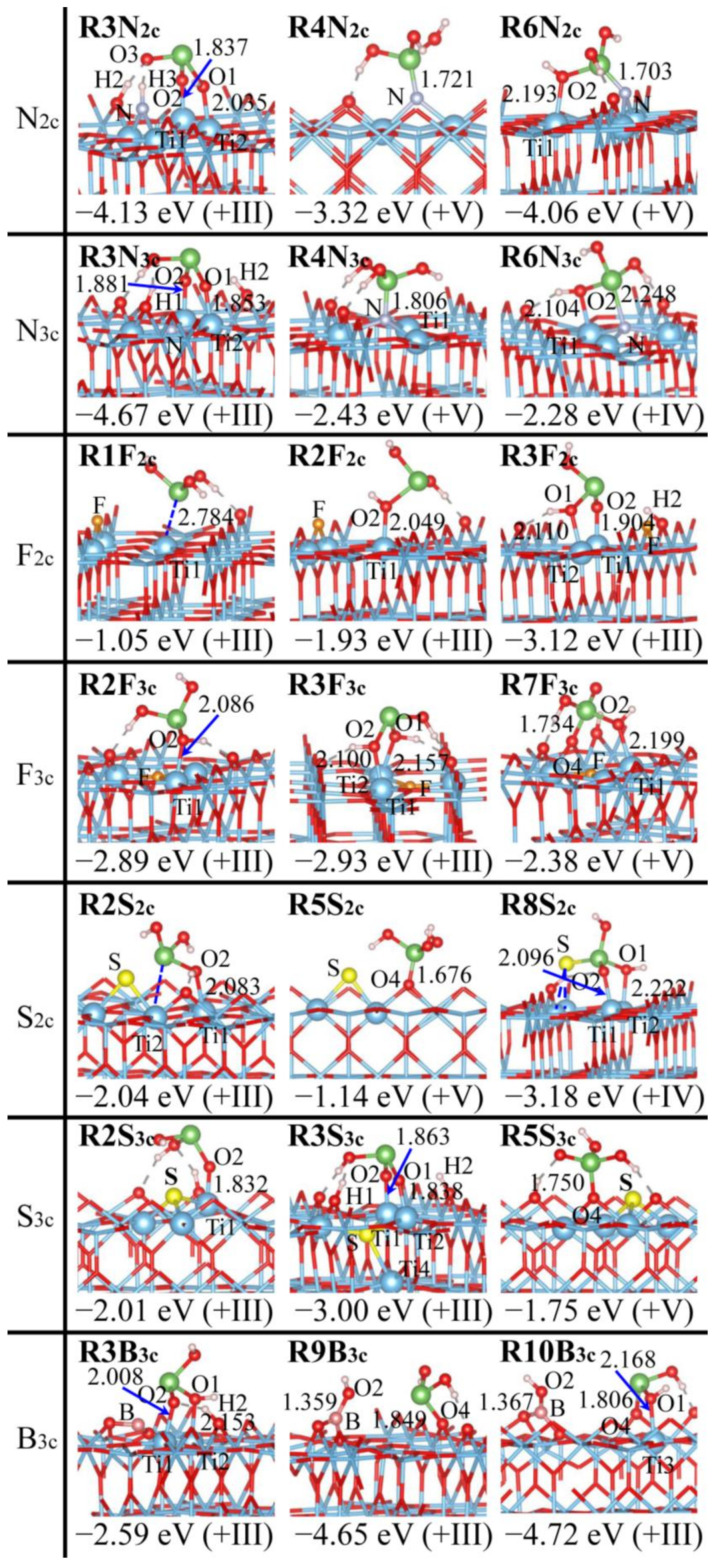
Adsorption configurations of As(OH)_3_ over rutile (110) surface with O_2c_, O_3c_ site being doped (D_O_ = N, F, S, B), together with adsorption energies (*E*_ad_) and oxidation states for the As centers (in parentheses). Color scheme: Ti (blue), O (red), As (green), H (white), N (gray), F (orange), S (yellow), and B (pink). Selected H-bonds are indicated by dashed gray lines. Distances are given in Å.

As(OH)_3_ adsorption over a rutile (110) surface with the O_2c_, O_3c_ site being doped (D_O_ = N, F, S, B), as shown in Figure 3 and Figure S6, is more perturbed than with the Ti_5c_ site being doped. N doping creates the additional adsorption sites [40,41], and As(OH)_3_ bonding to N_2c_ and N_3c_ generates three and two new adsorption structures, respectively. **R1**, **R2**, and **R3** remain similar to how they appear at a pristine surface, albeit with some differences: the Ti-As interaction is promoted in **R1N_2c_** (2.673 vs. 2.917 Å in **R1Pr**) and **R2N_3c_** (2.795 vs. 3.078 Å in **R2Pr**), while dual proton transfers occur in **R3N_2c_** and **R3N_3c_** instead of the single-proton transfer in **R3Pr**, and H3 in **R3N_2c_** bonds directly to N_2c_. The As-N bond is created in **R4N_2c_** and **R4N_3c_**, and a second bonding (Ti-O2) leads to bidentate **R6N_2c_** and **R6N_3c_**, while **R6N_3c_** is less favorable due to the formation of a four-membered ring (Ti1NAsO2) (see the *E*_ad_ in Figure 3). It is surprising to find that **R5N_2c_** with only an As-O4 bond exists stably (Figure S6).

F doping alters As(OH)_3_ adsorption less than N doping, and a majority of adsorption structures (**R1F_2c_**, **R2F_2c_**, **R3F_2c_**, **R2F_3c_**, and **R3F_3c_**) remain similar to how they appear at a pristine surface. The As-O_S_ bond is created in **R5F_2c_** and **R7F_3c_**, and the additional Ti1-O2 bond greatly stabilizes **R7F_3c_** (*E*_ad_ = −2.38 vs. −0.25 eV, Figure 3 and Figure S6). Owing to the close chemo-properties of S and O, **R1S_2c_**, **R2S_2c_**, and **R2S_3c_** are structurally similar and have a comparable *E*_ad_ as those at a pristine surface (Figure 3 and Figures S3 and S6). In addition to **R5S_2c_** and **R5S_3c_** with the As-O_S_ bond, **R4S_2c_** with the As-S bond is produced and exists stably. The large atomic radius causes S to protrude outwards and facilitates the interaction with As(OH)_3_, which further leads to a tridentate complex: **R8S_2c_** (Ti1-O2 + Ti2-O1 + As-S). B_2c_ doping is not considered due to its markedly lower stability compared to B_3c_ doping (5.17 eV), which is distinct from other dopants (e.g., 0.15 eV for N_2c_ vs. N_3c_ doping). B_3c_ doping causes the same surface rearrangement as B_5c_ doping (Figure S7). In addition to **R2B_3c_** and **R3B_3c_**, which are structurally similar to how they appear at a pristine surface, **R9B_3c_** and **R10B_3c_** appear and the As-O4 bond forms at expense of the As-O2 bond rupture. **R10B_3c_** is further stabilized by the Ti3-O1 bonding.

The adsorption configurations of As(OH)_3_ at the rutile (110) surface have the following stability trend: tridentate (**R8**) > bidentate (**R3**, **R6**, **R7**) > monodentate (**R2**, **R4**, **R5**) > physisorbed (**R1**) (see the *E*_ad_ in Figure 3 and Figure S6). **R3** is generally the most preferred: −3.12, −2.93, −3.18, −3.00, −4.13, and −4.67 eV for **R3F_2c_**, **R3F_3c_**, **R8S_2c_** (transformed barrierlessly from **R3S_2c_**), **R3S_3c_**, **R3N_2c_**, and **R3N_3c_**, respectively. This also suggests that doping the O_2c_, O_3c_ site may be more effective in enhancing As(OH)_3_ adsorption than doping the Ti_5c_ site (Figure 2). For B_3c_ doping, the preference of **R10B_3c_** over **R3B_3c_** (−4.72 vs. −2.59 eV) is ascribed to the structural reconstruction. This is confirmed by the superior stability of **R9B_3c_** (−4.65 eV) that is monodentate while it undergoes a similar reconstruction. Rutile (110) with doping of the O site is also efficient for As(OH)_3_ adsorption and removal, and the efficacy can be regulated within a wide range through the choice of dopants. The preferred adsorption with the O_3c_ vs. O_2c_ site being doped ranks as N > S > F, consistent with the likelihood to form more bonds: As(OH)_3_ forms a direct bond with N_3c_ in **R4N_3c_** and **R6N_3c_**, creating the tetra-coordinated N site, and S_2c_ in **R4S_2c_** and **R8S_2c_**, creating the tri-coordinated S site, whereas it forms no bond with F_2c_ and F_3c_. **R3N_3c_** and **R3F_2c_** are the most favorable for N and F doping, while due to the tridentate motif, **R8S_2c_** is slightly preferred over **R3S_3c_**. This is corroborated by the stability trend of adsorption structures with the As-O/As-D_O_ bond (N > S > F): for As-O4 bonding, **R5N_2c_** (−2.82 eV) > **R5S_2c_**, **R5S_3c_** (−1.14~−1.75 eV) > **R5F_2c_** (−0.25 eV), and for As-D_O_ bonding, **R4N_2c_** (−3.32 eV) > **R4S_2c_** (−0.85 eV) > **R4F_2c_** (non-existent).

### 2.3. As(OH)_3_ Adsorption by Doped Anatase (101)

**Figure 4:**
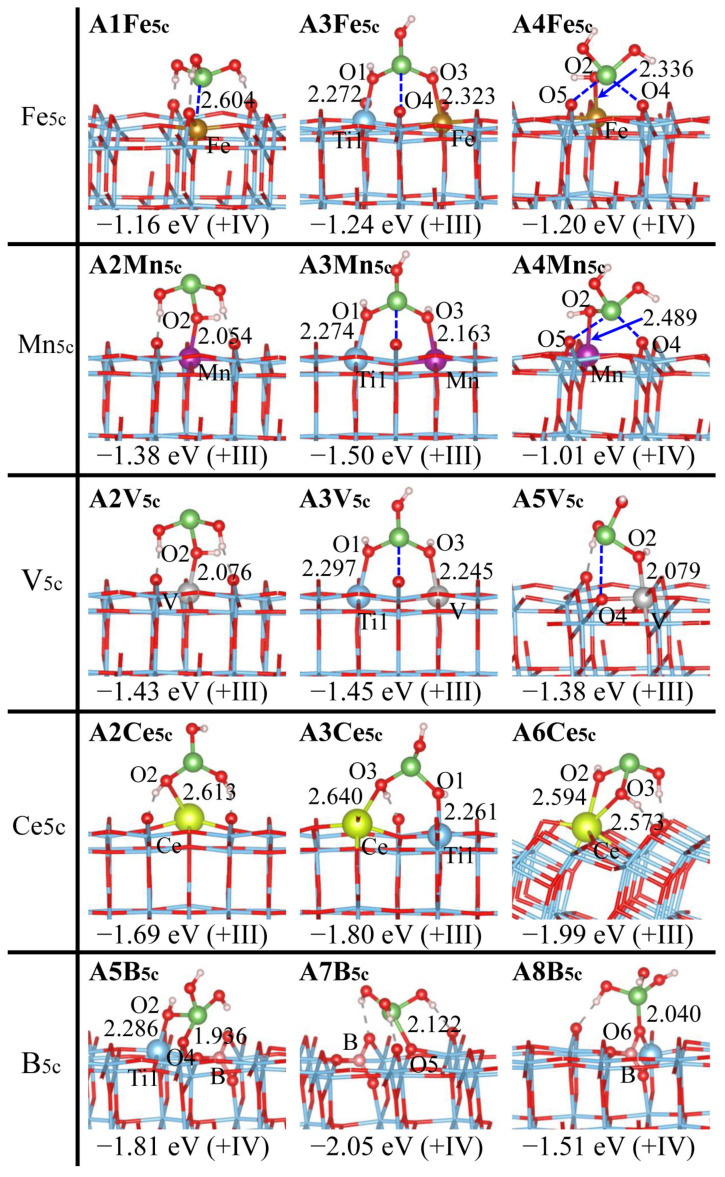
Adsorption configurations of As(OH)_3_ over the anatase (101) surface with the Ti_5c_ site being doped (D_Ti_ = Fe, Mn, V, Ce, B), together with adsorption energies (*E*_ad_) and oxidation states for the As centers (in parentheses). Color scheme: Ti (blue), O (red), As (green), H (white), Fe (golden), Mn (purple), V (silvery), Ce (yellow), and B (pink). Selected H-bonds are indicated by dashed gray lines. Distances are given in Å.

**Figure 5:**
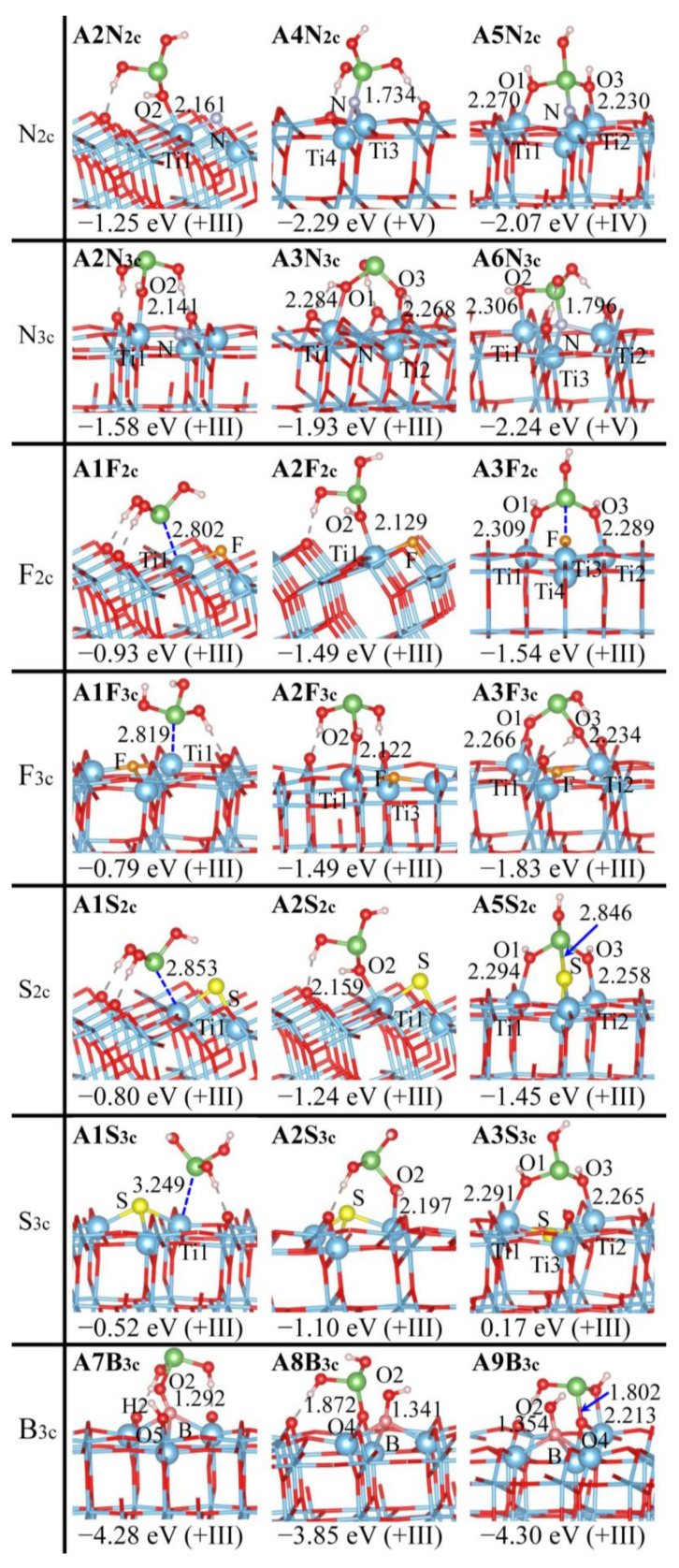
Adsorption configurations of As(OH)_3_ over the anatase (101) surface with the O_2c_, O_3c_ site being doped (D_O_ = N, F, S, B), together with adsorption energies (*E*_ad_) and oxidation states for the As centers (in parentheses). Color scheme: Ti (blue), O (red), As (green), H (white), N (gray), F (orange), S (yellow), and B (pink). Selected H-bonds are indicated by dashed gray lines. Distances are given in Å.

Figure 4, Figure 5 and Figures S8 and S9 depict the adsorption structures of As(OH)_3_ over the anatase (101) surface with the Ti_5c_, O_2c_, O_3c_ site being doped (D_Ti_ = Fe, Mn, V, Ce, B; D_O_ = N, F, S, B). Doping causes less alteration for As(OH)_3_ adsorption over anatase (101) vs. the rutile (110) surface, which can be deduced partially from the number of the disruption-prone physisorbed complexes (**A1** and **R1**): nine vs. three remain after doping. Particularly, F_2c_, F_3c_, and S_3c_ doping has a total of three adsorption structures (**A1**, **A2**, and **A3**) that are exactly identical to those at a pristine surface.

All new adsorption configurations are featured by the direct bonding of As(OH)_3_ with the doped sites, except B, while those with only the As-O_S_ bond vanish. This confirms less alteration at the anatase (101) than at the rutile (110) surface: (1) As(OH)_3_ adsorption is similar for Fe_5c_, Mn_5c_, and V_5c_ doping. The bidentate **A5** (D_Ti_-O2 + As-O4) is destabilized by the formation of a four-membered ring (D_Ti_O2AsO4), while in **A4**, the ring strain is alleviated where the As center forms the interaction with O4 and O5 instead of direct bonding. (2) Ce_5c_ and B_5c_ doping behaves distinctly due to a structural disruption (Figures S10 and S11), as discussed for rutile (110). However, **A6Ce_5c_** with two Ce-O_S_ bonds becomes preferred over **A3Ce_5c_** (Figure 4), probably due to the inferior reactivity of the Ti_5c_ sites at the anatase (101) surface [53,54]. Ce_5c_ doping enhances the reactivity, and the formation of more Ce-O_As_ bonds promotes adsorption (|*E*_ad_|: **A6Ce_5c_** > **A3Ce_5c_** > **A3Pr**), consistent with shorter Ce-O_As_ bonds in **A6Ce_5c_** vs. **A3Ce_5c_** (2.573, 2.594 vs. 2.640 Å). Although all of these have the As-O_2c_ bond, **A5B_5c_**, **A7B_5c_**, and **A8B_5c_** have distinct chemical environments for O_2c_: Ti-O_2c_-Ti, O_2c_ transformed from O_3c_, and B-O_2c_-Ti, respectively. (3) N_2c_ doping leads to monodentate **A4N_2c_** (As-N) and tridentate **A5N_2c_** (As-N + Ti1-O1 + Ti2-O3), while N_3c_ doping produces bidentate **A6N_3c_** (As-N + Ti1-O2). (4) S_2c_ doping results in tridentate **A5S_2c_** (Ti1-O1 + Ti2-O3 + As-S), in addition to bidentate **A6S_2c_** (Ti1-O2 + As-S) that is disfavored by the large Ti-S distances (ca. 2.665 vs. 2.349 Å in **A1S_2c_**) (Figure 5 and Figure S9). (5) B_3c_ doping causes the structural rearrangement producing three Ti-B bonds (Figure S12). **A7B_3c_** has a B-O_As_ bond, as further stabilized by the H2 transfer to O5, while **A4B_3c_** has an As-B bond and **A8B_3c_** has an As-O_S_ bond, and a second bonding (Ti-O_As_) produces **A6B_3c_** and **A9B_3c_**.

The stability of different adsorption configurations generally declines as tridentate (**A5**), bidentate (**A3**, **A6**, **A9**) > monodentate (**A2**, **A4**, **A8**) > physisorbed (**A1**) (see Figure 4, Figure 5 and Figures S8 and S9). The most favorable adsorption configurations may not be **A3**, and have *E*_ad_ values of −1.24 eV for **A3Fe_5c_**, −1.50 eV for **A3Mn_5c_**, −1.45 eV for **A3V_5c_**, −1.99 eV for **A6Ce_5c_**, −2.05 eV for **A7B_5c_**, −2.29 eV for **A4N_2c_**, −2.24 eV for **A6N_3c_**, −1.54 eV for **A3F_2c_**, −1.83 eV for **A3F_3c_**, −1.45 eV for **A5S_2c_**, −1.10 eV for **A2S_3c_**, and −4.30 eV for **A9B_3c_**. These values agree with the literature reports available: the Ce-Ti hybrid oxide shows enhanced adsorption for As(III) compared to pure TiO_2_ [55], and Pb^2+^ adsorption by anatase increases due to F doping [42]. The choice of different dopants causes the As(OH)_3_ adsorption efficiency to vary within a wide range, and doping the O_2c_, O_3c_ rather than the Ti_5c_ site may exhibit larger promoting effects for As(OH)_3_ adsorption, which is in line with the results of the rutile (110) surface. However, the doped anatase (101) surface is apparently less efficient for As(OH)_3_ adsorption and removal than the doped rutile (110) surface, implying the critical role of crystal control played therein. Albeit both being bidentate, **A9B_3c_** is preferred over **A6B_3c_** due to the B-O2H bond formation, as verified by the superior stability of **A8B_3c_** that has B-O2H bonding, although it is monodentate. In order to form the tridentate motif, **A5N_2c_** has the stretched As-O1 and As-O3 bonds (ca. 1.880 Å), which lead to the lower stability compared to **A4N_2c_**. **A7B_5c_** has As-O_2c_ bonding similar to **A5B_5c_** and **A8B_5c_**, while it is preferred due to the high reactivity of its O_2c_ site that evolves from O_3c_. Albeit being the bidentate motif, **A3S_3c_** is destabilized by a serious structural distortion: the strong electrostatic repulsion with O1 and O3 causes the S atom to be pushed below the top surface, and the Ti1-Ti2 distances are significantly enlarged (4.387 vs. 3.815 Å in **A3Pr**).

### 2.4. Regulatory Mechanism of As(OH)_3_ Adsorption by Doping

The oxidation states of As in various As(OH)_3_ adsorption structures, as determined by the Bader charge and magnetic moments (Figures S13–S16), are listed in Figure 2, Figure 3, Figure 4, Figure 5 and Figures S2, S6, S8 and S9. As(III), As(IV), and As(V) correspond to Bader charges of 1.51~1.70, 1.78~2.06, and 2.24~2.55 |e|, respectively [31,56,57]. As(IV) differs from As(III) and As(V) by having the unpaired electrons and noticeable magnetic moments [6,58,59,60]. The assignment of oxidation states is further validated by reference compounds, and the Bader charge of the As centers in As_2_O_3_ and As_2_O_5_ amounts to 1.53 and 2.25 |e|, respectively. The As centers in the adsorption configurations are often in +III form, while they are automatically oxidized to As(IV) or As(V) when forming direct bonds with the highly electronegative surface O and N atoms. These are distinct from pristine surfaces, where only the As(III) form is detected (Figure S3) [31]. The doped rutile (110) rather than anatase (101) surface is more ready to execute As(III) oxidation to As(V): nine (**R5B_5c_**, **R7B_5c_**, **R4N_2_**_c_, **R5N_2c_**, **R6N_2c_**, **R5S_2c_**, **R4N_3c_**, **R7F_3c_,** and **R5S_3c_**) vs. two (**A4N_2c_** and **A6N_3c_**). The +IV species is prone to occur when As(OH)_3_ is multidentately adsorbed: **R5Fe_5c_**, **R5Mn_5c_**, **R5V_5c_**, **R6N_3c_**, **R5F_2c_**, **R4S_2c_**, **R8S_2c_**, **A5Fe_5c_**, **A5Mn_5c_**, **A5B_5c_**, **A7B_5c_**, **A8B_5c_**, **A5N_2c_**, and **A6S_2c_**. Owing to the strong interaction among the As center and two O_S_ atoms, **A4Fe_5c_** and **A4Mn_5c_** have the As(IV) species. Instead, **A4V_5c_** has the apparently larger As-O_S_ distances (ca. 2.90 Å), and hence the As center remains to be in the +III state. As(IV) may even appear when the As centers form the interaction with the Ti_5c_ site: **R2Mn_5c_**, **A1Fe_5c_**, **A1N_3c_**, and **A1B_5c_**. All adsorption structures with As(V) have superior stability, and As(IV), rarely found in natural environments, is stabilized pronouncedly at the doped TiO_2_ surfaces; **R8S_2c_** and **A7B_5c_** become the most preferred for S_2c_ and B_5c_ doping.

Doping alters the electronic structures of TiO_2_ and further As(OH)_3_ adsorption [61] (see the charge density difference and spin density isosurfaces for Fe_5c_, N_2c_, and N_3c_ doping in Figures S17 and S18). Fe doping is used to illustrate the change in charged states during doping of the TiO_2_ surfaces (see Figure S19). Charge transfer occurs from Fe to the adjacent O atoms, and the Bader charge of Fe shows some increase. Note that Fe(OH)_3_ is used as a reference for Fe in the +III state. The alteration of charged states due to Fe doping is a localized behavior, and one or several adjacent Fe-bonded O atoms fall between the O^2−^ and O^•−^ states, while the rest of the atoms of the TiO_2_ surfaces remain nearly intact. Similar changes can be found for other dopants. Accordingly, the doped sites and proximal-O atoms undertake the change in charged states due to doping [35,36,37,38,39,40,41,42,43]. Electron transfer occurs considerably between the As(OH)_3_ and TiO_2_ surface and is promoted by the direct interaction of the As center with the doped site that further leads to As(III) oxidation. In addition to the doped site, the adjacent Ti and O atoms participate closely during As(OH)_3_ oxidation (Figures S13–S16), e.g., O4 in **R5Fe_5c_** in O^•−^ form and subsurface Ti3 in **R5N_2c_** in Ti(III)^•^ form, consistent with the XPS spectra indicating that doping renders the Ti atoms to be more electronegative [61]. Electron redistribution and dispersion greatly stabilize the As species, especially As(IV), where more atoms may be involved, e.g., at least six O atoms in **A4Fe_5c_** have clear spin densities and fall between O^2−^ and O^•−^. Electron back-donation from TiO_2_ to As(OH)_3_ is promoted due to the formation of the multidentate motif, and As(V) is likely to be re-reduced to As(IV), e.g., Ti1 in **R4N_3c_** is in Ti(III)^•^ form, while it turns to Ti(IV) in **R6N_3c_** due to the Ti1-O2 bonding that causes As(V) re-reduction.

**Figure 6:**
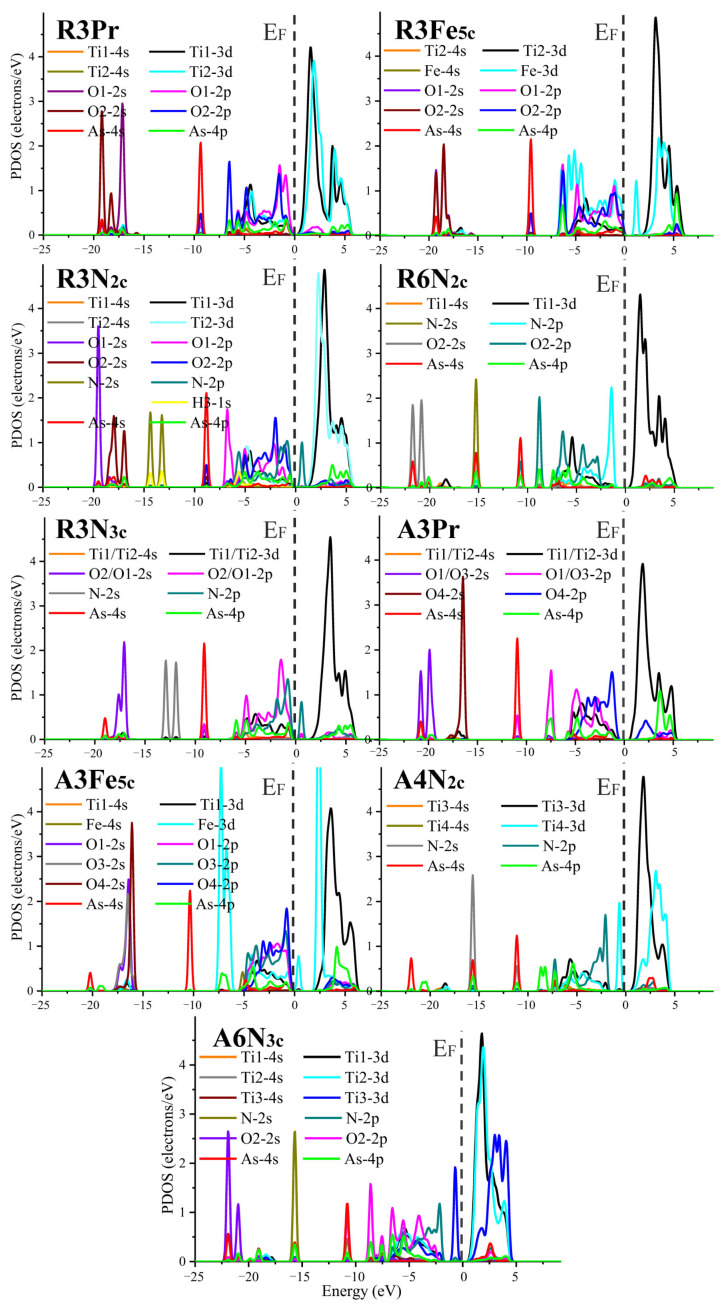
Projected density of states (PDOS) for As(OH)_3_ adsorption at rutile (110) and anatase (101) surfaces in pristine and doped forms (D_Ti_ = Fe; D_O_ = N). The Fermi level (E_F_) is set to zero energy and highlighted by gray dotted line.

The PDOS peaks for atoms that are closely involved during As(OH)_3_ adsorption are significantly affected. In all scenarios (pristine and doped at the Ti_5c_, O_2c_, and O_3c_ sites), only specific valence orbitals have considerable PDOS overlapping and play a central role during As(OH)_3_ adsorption, e.g., Ti-*3d*, O-*2p*, As-*4p*, Fe-*3d*, and N-*2p* instead of Ti-*4s*, O-*2s*, As-*4s*, Fe-*4s*, and N-*2s* (see Figure 6 and Figure S20). The major PDOS peaks fall at the right side of the Fermi level for Ti-*3d* (e.g., 0.2~5.9 eV in **R3Pr**, 1.7~6.2 eV in **R3Fe_5c_**, and 0.4~5.3 eV in **R6N_2c_**) and Fe-*3d* (e.g., 0.7~5.9 eV in **R3Fe_5c_**), whereas they fall at the left side for O-*2p* (e.g., −6.9~0.0 eV in **R3Pr**, −6.9~0.1 eV in **R3Fe_5c_**, and −9.5~−1.0 eV in **R6N_2c_**), As-*4p* (e.g., −6.9~−0.2 eV in **R3Pr**, −6.9~0.1 eV in **R3Fe_5c_**, and −9.2~−1.0 in **R6N_2c_**) and N-*2p* (e.g., −7.9~−0.6 eV in **R6N_2c_**). For the pristine rutile (110) surface, O-*2p* has the PDOS overlapping with the secondary peak of Ti-*3d* (e.g., −6.8~−0.1 eV in **R3Pr**), and this situation remains for Fe_5c_ doping, e.g., overlapping with the secondary PDOS peaks of Ti-*3d* (−6.7~0.2 eV) and Fe-*3d* (−7.0~0.2 eV) in **R3Fe_5c_**. However, the major PDOS peaks of O-*2p*, As-*4p*, and N-*2p* have nearly identical PDOS domains that are beneficial for As(OH)_3_ adsorption, as evidenced by the As-N and As-O_S_ bonding and large |*E*_ad_|. Although with similar bonding mechanisms of rutile (110) as As(OH)_3_, anatase (101) may have the obviously weaker PDOS peaks for Ti-*3d* (secondary), Fe-*3d* (tertiary), and N-*2p* (major) that disfavor adsorption, e.g., −5.4~−0.2 eV for Ti-*3d* and −5.4~−0.1 eV for Fe-*3d* in **A3Fe_5c_**. The PDOS overlapping of O1-*2p* with Ti1-*3d* and O3/O4-*2p* with Fe-*3d* shifts towards the higher-energy domains (−5.0~−0.2 vs. −5.7~−0.9 eV in **A3**), and neither of the two largest PDOS peaks of Fe-*3d* (1.8~6.3 eV; −7.9~−6.0 eV) participate during the interaction with those of O-*2p*. The major PDOS peak of N-*2p* in **A4N_2c_** is further divided into two sub-domains, −7.7~−4.4 and −4.4~−1.6 (main) eV, and the former dominates the overlapping with those of As-*4p*. This can be interpreted as that whether for pristine or for Fe-, N-doped forms, rutile (110) is more efficient than anatase (101) for As(OH)_3_ adsorption.

## 3. Computational Section

### 3.1. Models

The optimized lattice parameters of TiO_2_ polymorphs amount to a = b = 4.608 Å, c = 2.973 Å for rutile and a = b = 3.793 Å, c = 9.594 Å for anatase, and show good agreement with the literature reports [62,63]; (110) and (101) stand for the most frequently exposed and extensively studied facets for rutile and anatase [53,64], and their models are stoichiometric with the molecular formulas of Ti_60_O_120_ and Ti_48_O_96_, respectively (Figure 1). All models consist of six atomic layers [65,66,67], and the slabs were separated by 20.000 Å to avoid image interactions. The effects of water at TiO_2_ surfaces on As(OH)_3_ adsorption [68] were further investigated and found to be slight (see more details in Supplementary Materials S1: Effects of Adsorbed Water).

Figure 1 shows that the TiO_2_ surfaces have 2-fold (O_2c_) and 3-fold (O_3c_) coordinated O and 5-fold (Ti_5c_) and 6-fold (Ti_6c_) coordinated Ti atoms. The dopants investigated presently included D_Ti_ = Fe, Mn, V, Ce, B at the Ti_5c_ site and D_O_ = N, F, B, S at the O_2c_, O_3c_ site [26,27,30,31,32,36,37,38,39,40,41,42,43,61]. The As(OH)_3_ adsorption configurations of rutile (110) and anatase (101) were nominated to be **RnD_Ti_D_O_** and **AnD_Ti_D_O_**, where R, A, D_Ti_, and D_O_ stand for rutile (110), anatase (101), and dopants at the Ti and O sites, and n refers to the number of specific adsorption configurations. For instance, **R2Fe_5c_** and **A4N_2c_** refer to the No. 2 adsorption configuration at the rutile (110) surface with Fe_5c_ doping and the No. 4 adsorption configuration at the anatase (101) surface with N_2c_ doping.

### 3.2. Methods

Spin-polarized DFT calculations were conducted using the Perdew–Burke–Ernzerhof (PBE) exchange–correlation functional at the generalized gradient approximation (GGA) level, as implemented within the Vienna ab initio simulation package (VASP) [69,70]. The projected-augmented wave (PAW) approach was employed to handle the electron–ion interactions, and the non-covalent interactions were described by the standard DFT-D3 (BJ) scheme [71,72]. DFT + U was adopted for the on-site Coulomb interaction of 3*d* and 4*f* electrons [73], and Hubbard correction was recommended for Ti-3*d* (U*_eff_* = 4.2 eV) [74,75], Fe-3*d* (U*_eff_* = 5.0 eV) [76], Mn-3*d* (U*_eff_* = 4.5 eV) [77,78], V-3*d* (U*_eff_* = 3.0 eV) [79,80], and Ce-4*f* (U*_eff_* = 5.0 eV) [77,78]. The Brillouin zone was sampled using the 2 × 2 × 1 Monkhorst-Pack grid [31,56]. The cut-off energy was set to 400.0 eV and validated by higher values such as 450.0 and 500.0 eV. The structural optimizations were converged when the forces acting on all atoms were less than 0.05 eV/Å.

The adsorption energies (*E*_ad_) of As(OH)_3_ over the pristine and doped TiO_2_ surfaces were calculated similarly:

*E*_ad_(pristine) = *E*_As(OH)3/TiO2_ − (*E*_TiO2_ + *E*_As(OH)3_) (1)

*E*_ad_(doped) = *E*_As(OH)3/D-TiO2_ − (*E*_D-TiO2_ + *E*_As(OH)3_) (2)

where *E*_As(OH)3_, *E*_TiO2_, and *E*_D-TiO2_ stand for the electronic energies of As(OH)_3_ and pristine and doped TiO_2_ surfaces, while *E*_As(OH)3/TiO2_ and *E*_As(OH)3/D-TiO2_ refer to the electronic energies of As(OH)_3_ adsorption structures corresponding to the pristine and doped TiO_2_ surfaces, respectively.

To further the understanding of interactions between As(OH)_3_ and TiO_2_ surfaces and effects of doping and facet control onto As(OH)_3_ adsorption, Bader charge and spin density calculations were conducted [81], and the oxidation states of the As centers in adsorption configurations were then determined. The isosurfaces of spin densities were visualized by means of the VESTA 3 software [82]. In addition, the PDOS was calculated to gain insight into the bonding mechanisms between As(OH)_3_ and TiO_2_ surfaces [83].

## 4. Conclusions

This study presents a comprehensive understanding of how doping regulates As(III) adsorption and removal by TiO_2_ and the critical roles of crystal control played therein. As(OH)_3_ adsorption is more altered at the rutile (110) rather than anatase (101) surface. (1) All dopants except B_5c_, F_2c_, and F_3c_ form direct bonding with As(OH)_3_ that can be detected in a majority of new adsorption structures. (2) The atomic radius is critical for adsorption when doping the Ti_5c_ site (Ce >> Ti, Fe, Mn, V >> B). Ce_5c_ and B_5c_ doping may have distinct As(OH)_3_ adsorption from Fe_5c_, Mn_5c_, and V_5c_ doping: Ce_5c_ doping leads to the bidentate mononuclear **R6Ce_5c_** and **A6Ce_5c_**, while B_5c_ doping transforms O_3c_ to O_2c_ that further forms a direct bond with As(OH)_3_ in **R7B_5c_** and **A7B_5c_**. (3) For doping the O_2c_, O_3c_ site, the perturbation extent of As(OH)_3_ adsorption is N > S > F and accords with the likelihood of forming more bonds. N doping stabilizes **R5N_2c_** with only the As-O_S_ bonding and has the largest potential to form the As-N bond, with production of monodentate (**R4N_2c_**, **R4N_3c_**, and **A4N_2c_**), bidentate (**R6N_2c_**, **R6N_3c_**, and **A6N_3c_**) and tridentate (**A5N_2c_**) motifs. S doping facilitates the interaction with As(OH)_3_ and leads to tridentate **R8S_2c_** and **A5S_2c_**. (4) Doping the O_2c_, O_3c_ rather than the Ti_5c_ site causes more structural perturbation and diversity to As(OH)_3_ adsorption.

As(OH)_3_ adsorption structures generally have the following stability trend: tridentate, bidentate > monodentate > physisorbed. Similar to the scenario of pristine TiO_2_ surfaces, **R3** and **A3** with two Ti-O_As_ bonds often remain to be the most preferred adsorption configurations, while this is not the case for B_5c_ (Ti-O_As_ + As-O_Ti_), S_2c_ (Ti-O_As_ + Ti-O_As_ + As-S) and B_3c_ (As-O_Ti_) doping of rutile (110), and Ce_5c_ (Ce-O_As_ + Ce-O_As_), B_5c_ (As-O_Ti_), N_2c_ (As-N), S_2c_ (Ti-O_As_ + Ti-O_As_ + As-S), N_3c_ (Ti-O_As_ + As-N), S_3c_ (Ti-O_As_), and B_3c_ (As-O_Ti_) doping of anatase (101). Doping is crystal-dependent, and the doped rutile (110) rather than anatase (101) surface is apparently more efficient for As(III) adsorption. In addition, doping the O_2c_, O_3c_ rather than the Ti_5c_ site may lead to larger promoting effects for adsorption, and the *E*_ad_ reaches −4.17, −4.13, and −4.67 eV for Mn_5c_, N_2c_, and N_3c_ doping of rutile (110) and −1.99, −2.29, and −2.24 eV, for Ce_5c_, N_2c_, and N_3c_ doping of anatase (101), respectively.

Distinct from pristine TiO_2_ surfaces, where the As centers are always in +III form, doping is prone to cause As(III) auto-oxidation when the As centers interact directly with the TiO_2_ surfaces. Rutile rather than anatase is more ready to trigger the auto-oxidation of As(III) to As(V). All adsorption structures with As(V) have superior stability, and doping also stabilizes the As(IV) species: **R8S_2c_** and **A7B_5c_** become the most preferred for S_2c_ and B_5c_ doping. The multidentate adsorption of As(OH)_3_ causes electron back-donation and may cause As(V) re-reduction to As(IV). The mechanisms regarding how doping regulates As(III) adsorption by TiO_2_ and the critical roles of crystal control played therein are further rationalized at the molecular level.

## Data Availability

Data are contained within this article and the Supplementary Materials.

## Supplementary Materials

The following supporting information can be downloaded at https://www.mdpi.com/article/10.3390/molecules29173991/s1.
