# Supplementary material for: How Doping Regulates As(III) Adsorption at TiO2 Surfaces: A DFT + U Study
Source: Molecules. 2024 Aug 23;29(17):3991. doi: 10.3390/molecules29173991 (PMC11396678; doi:10.3390/molecules29173991)
Supplement: Supplementary file 1 [file molecules-29-03991-s001.zip › molecules-3119447-supplementary.pdf]

S1. Effects of Adsorbed Water

The optimal adsorption configurations of $As(OH)_3$ over Fe_{5c}- and N_{3c}-doped rutile (110) surfaces (i.e., **R5Fe$_{5c}$** and **R3N$_{3c}$**) are chosen to investigate the effects of adsorbed water. It is assumed that when H_2O is more distant from $As(OH)_3$, it exerts the less influences onto $As(OH)_3$ adsorption. As shown in Figure S1, one H_2O molecule is placed at the Ti_{5c} site that is the nearest to $As(OH)_3$, and forms H-bond with $As(OH)_3$: $O_wH_w \cdots O2$ in **R5Fe$_{5c}$** (1.879 Å) and **R3N$_{3c}$** (1.851 Å). Nonetheless, the adsorption structures of $As(OH)_3$ remain nearly intact, and the Fe-O2 and As-O4 bonds in **R5Fe$_{5c}$** and the Ti1-O2 and Ti2-O1 bonds in **R3N$_{3c}$** vary not more than 0.071 Å due to H_2O presence.

The adsorption energy (E_{ad}) of $As(OH)_3$ over rutile (110) surfaces with presence of one proximal H_2O molecule is defined similarly as in absence of H_2O,

$$E_{ad} = E_{As(OH)3/TiO2\text{-}H2O} - (E_{TiO2\text{-}H2O} + E_{As(OH)3}) \quad (S1)$$

where $E_{As(OH)3}$ and $E_{TiO2\text{-}H2O}$ stand for electronic energies of $As(OH)_3$ and TiO_2 surface with presence of one H_2O molecule, and $E_{As(OH)3/TiO2\text{-}H2O}$ refer to the adsorption configuration of $As(OH)_3$ over TiO_2 surface with presence of one proximal H_2O molecule.

The E_{ad} values amount to −2.48 and −4.72 eV for **R5Fe$_{5c}$** and **R3N$_{3c}$** and are very close to those in absence of water (−2.54 and −4.67 eV), which accord well with the consistent adsorption structures of $As(OH)_3$ discussed above. Hence, adsorbed water may affect $As(OH)_3$ adsorption over TiO_2 surfaces but the influences are limited.

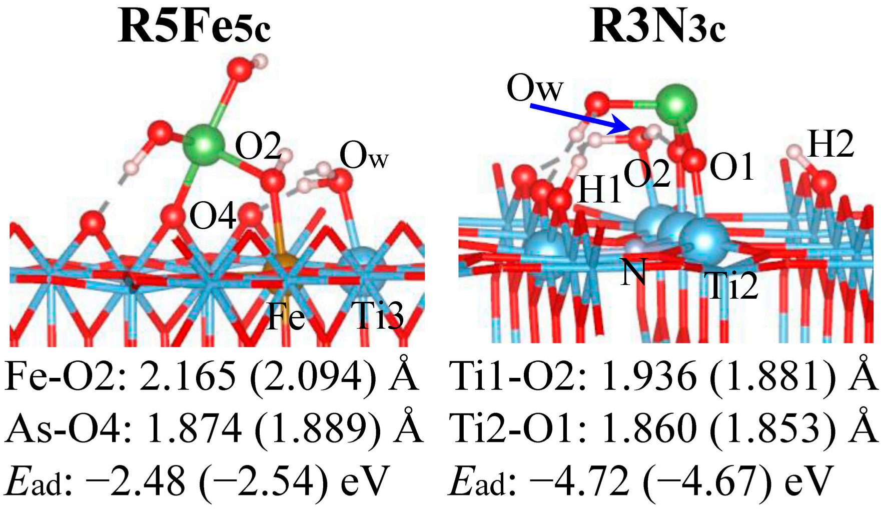

Figure S1. Selected adsorption configurations of $As(OH)_3$ over Fe_{5c}- and N_{3c}-doped rutile (110) surfaces with co-adsorption of one proximal H_2O molecule (**R5Fe5c** and **R3N3c**, the most stable respectively for Fe and N doping), together with bond distances and adsorption energies (E_{ad}). Color scheme: Ti (blue), O (red), As (green), H (white), Fe (golden), N (gray). Bond distances and adsorption energies (E_{ad}) with absence of H_2O molecules are listed in parentheses, and H-bonds are indicated by dashed gray lines.

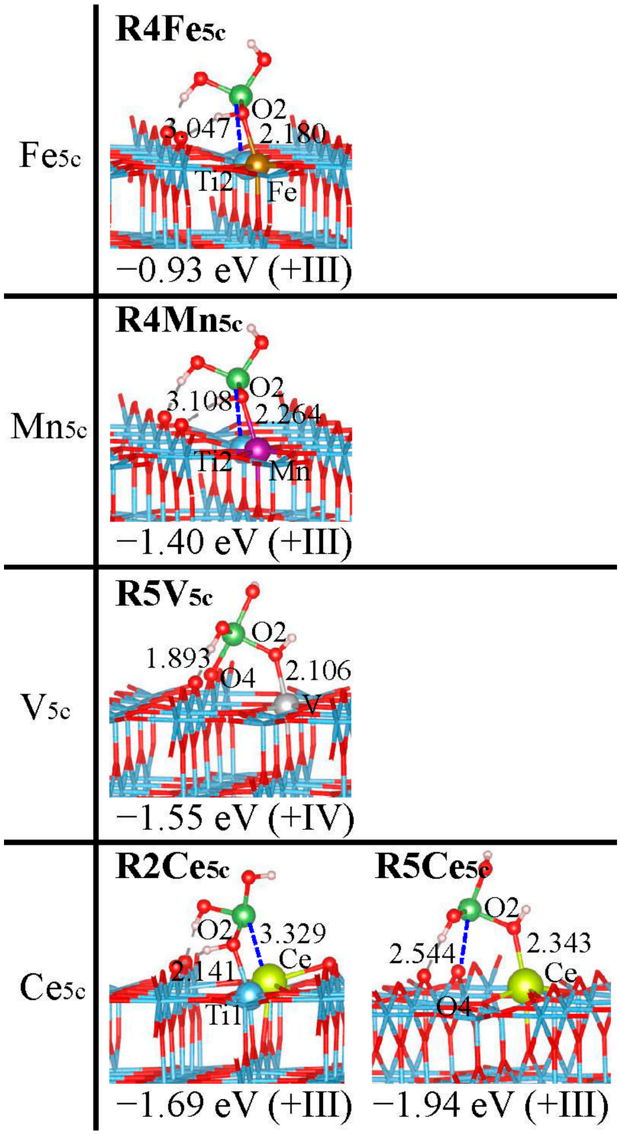

Figure S2. Less preferred adsorption configurations of $As(OH)_3$ at rutile (110) surface with the Ti_{5c} site being doped (D_{Ti} = Fe, Mn, V, Ce), together with adsorption energies (E_{ad}) and oxidation states for the As centers (in parentheses).

Color scheme: Ti (blue), O (red), As (green), H (white), Fe (golden), Mn (purple), V (silvery), Ce (yellow). Selected H-bonds are indicated by dashed gray lines. Distances are given in Å.

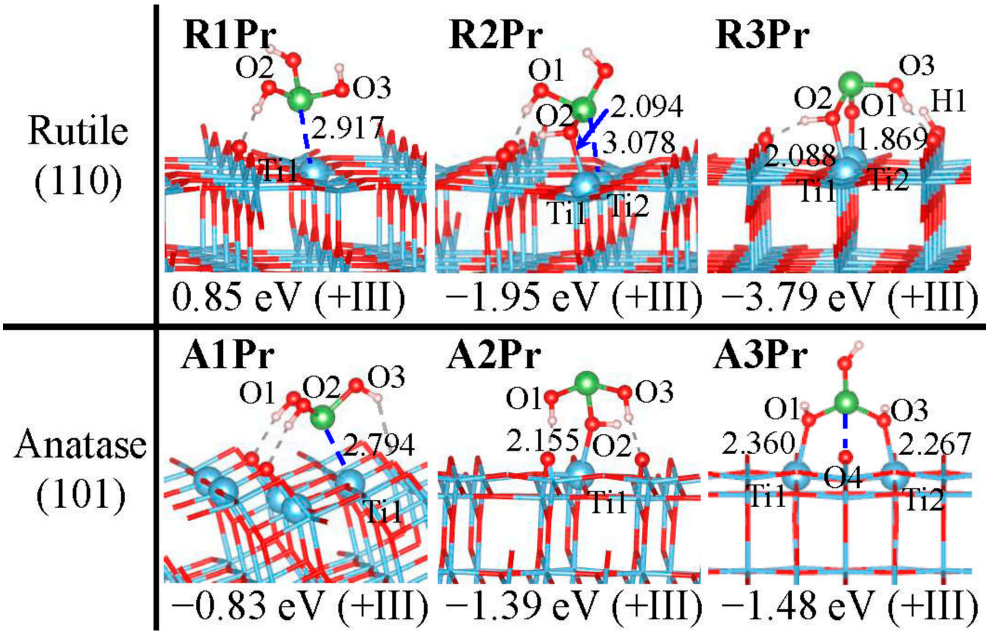

Figure S3. Adsorption configurations of $As(OH)_3$ at pristine surfaces, together with adsorption energies (E_{ad}) and oxidation states for the As centers (in parentheses).

Color scheme: Ti (blue), O (red), As (green), H (white). Selected H-bonds are indicated by dashed gray lines. Distances are given in Å.

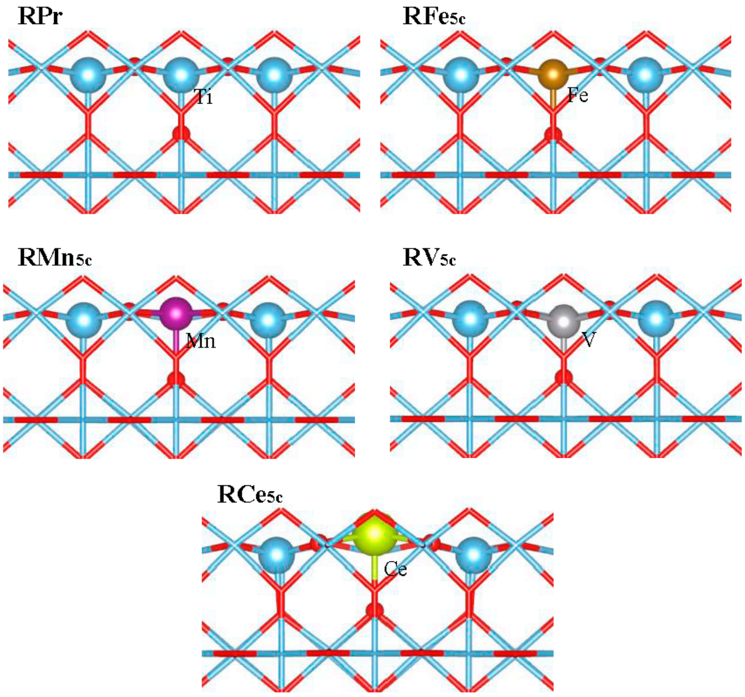

Figure S4. Side-views of rutile (110) surface in the pristine form and with the Ti_{5c} site being doped (D_{Ti} = Fe, Mn, V, Ce).

Color scheme: Ti (blue), O (red), H (white), Fe (golden), Mn (purple), V (silvery), Ce (yellow).

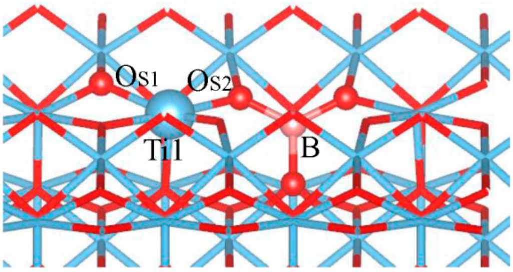

Figure S5. Local structure of rutile (110) surface with the Ti_{5c} site being doped by B, where the Ti-O_{S1} and Ti-O_{S2} bond distances are calculated to be approximately 2.028 and 2.065 Å instead of 1.948 and 1.948 Å at pristine surface.

Color scheme: Ti (blue), O (red), B (pink).

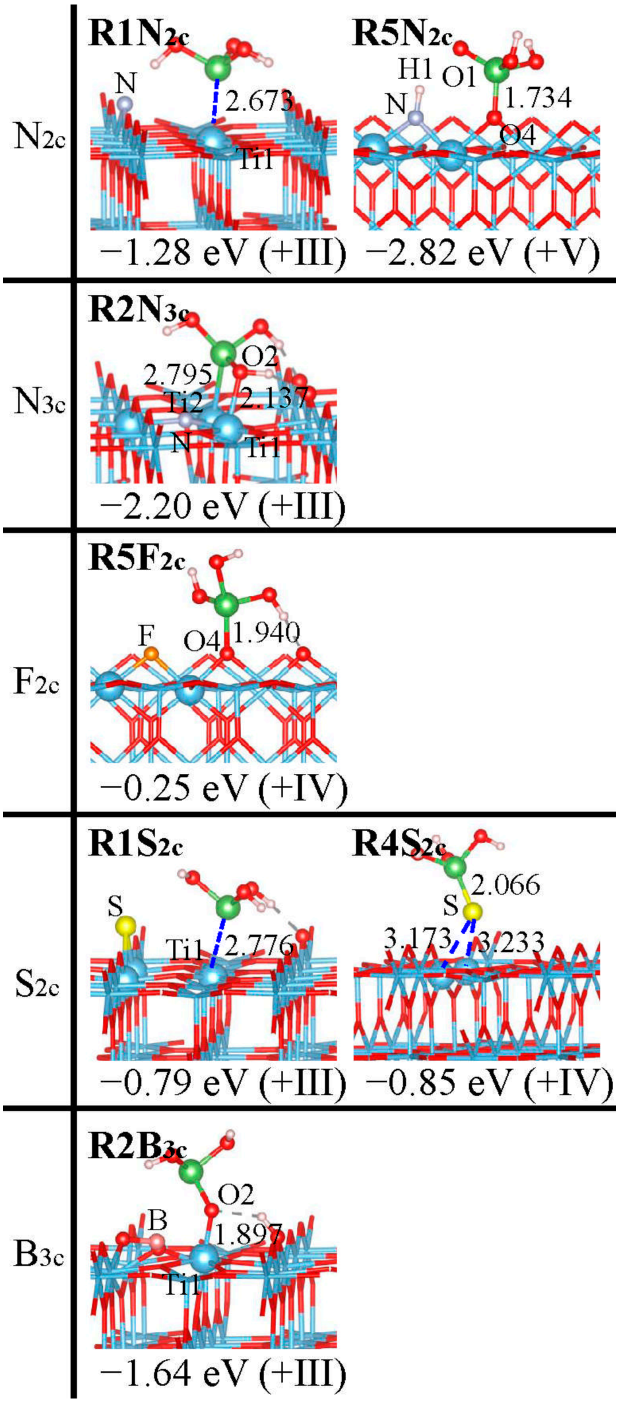

Figure S6. Less preferred adsorption configurations of $As(OH)_3$ at rutile (110) surface with the O_{2c}, O_{3c} site being doped (D_O = N, F, S, B), together with adsorption energies (E_{ad}) and oxidation states for the As centers (in parentheses).

Color scheme: Ti (blue), O (red), As (green), H (white), N (gray), F (orange), S (yellow), B (pink). Selected H-bonds are indicated by dashed gray lines. Distances are given in Å.

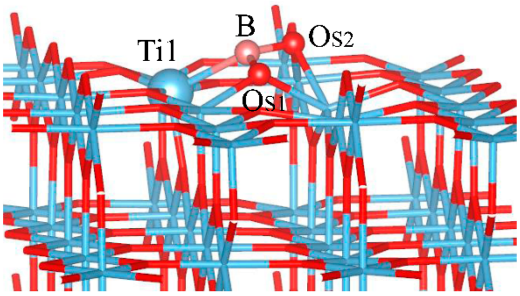

Figure S7. Local structure of rutile (110) surface with the O_{3c} site being doped by B, where B forms three direct bonds with surface: B-O_{S1} = 1.402 Å, B-O_{S2} = 1.376 Å, B-Ti1 = 2.274 Å. Color scheme: Ti (blue), O (red), B (pink).

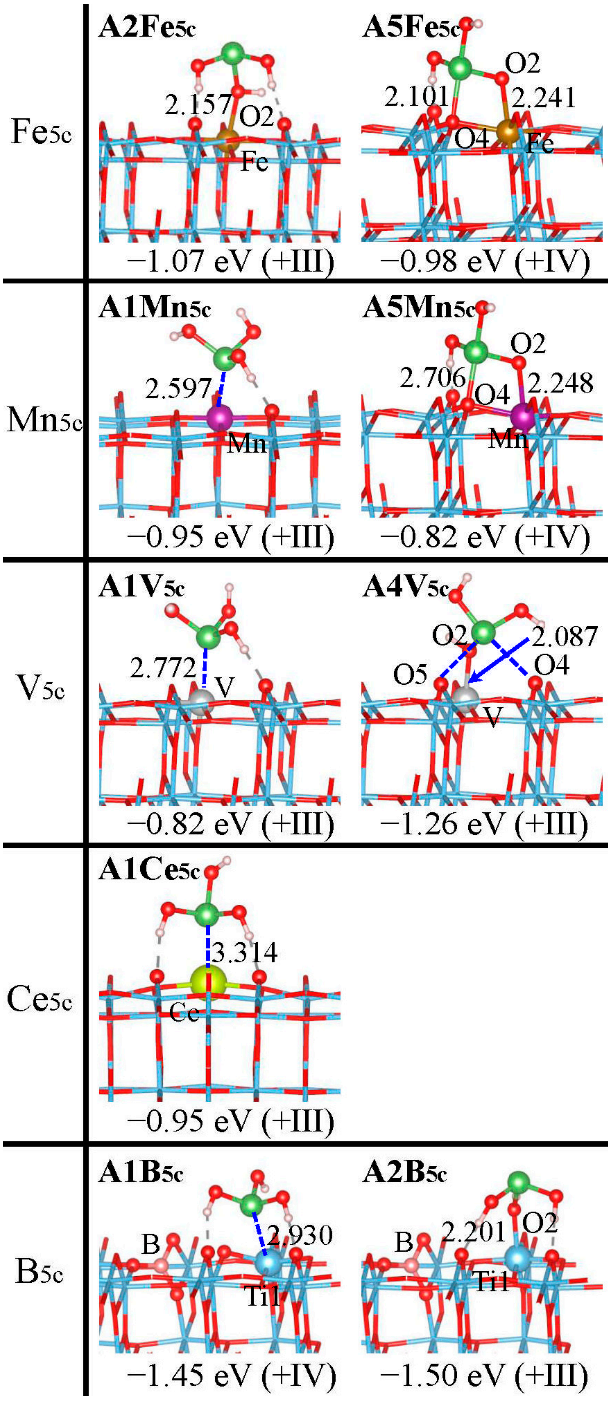

Figure S8. Less preferred adsorption configurations of $As(OH)_3$ at anatase (101) surface with the Ti_{5c} site being doped (D_{Ti} = Fe, Mn, V, Ce, B), together with adsorption energies (E_{ad}) and oxidation states for the As centers (in parentheses).

Color scheme: Ti (blue), O (red), As (green), H (white), Fe (golden), Mn (purple), V (silvery), Ce (yellow), B (pink). Selected H-bonds are indicated by dashed gray lines. Distances are given in Å.

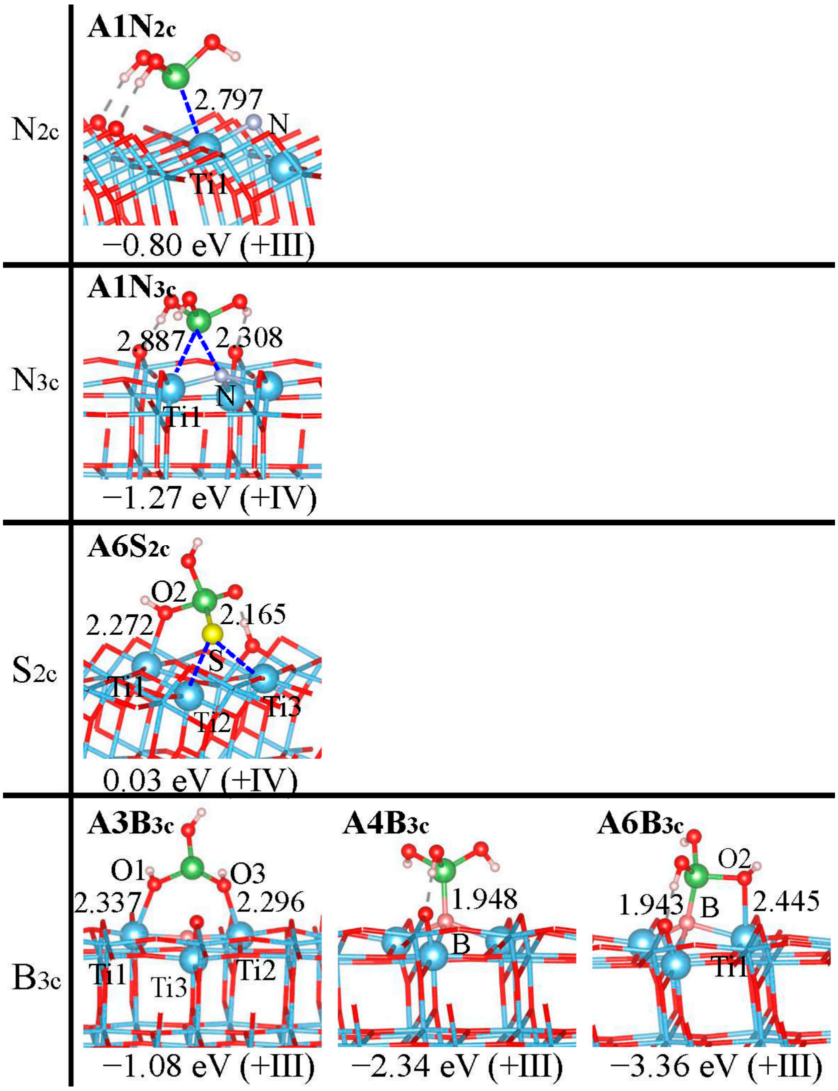

Figure S9. Less preferred adsorption configurations of $As(OH)_3$ at anatase (101) surface with the O_{2c}, O_{3c} site being doped (D_O = N, S, B), together with adsorption energies (E_{ad}) and oxidation states for the As centers (in parentheses).

Color scheme: Ti (blue), O (red), As (green), H (white), N (gray), S (yellow), B (pink). Selected H-bonds are indicated by dashed gray lines. Distances are given in Å.

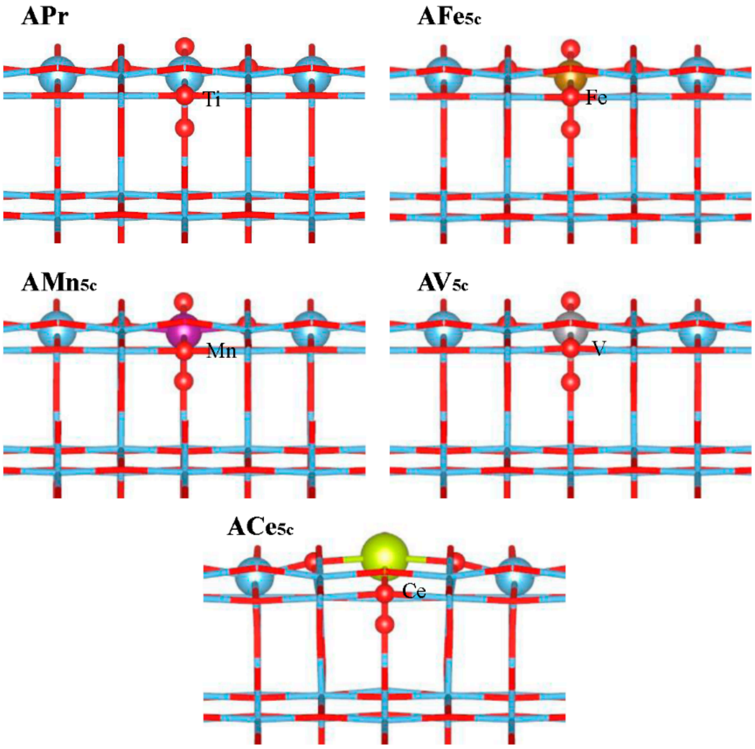

Figure S10. Side-views of anatase (101) surface in pristine form and with the Ti_{5c} site being doped (D_{Ti} = Fe, Mn, V, Ce).
Color scheme: Ti (blue), O (red), H (white), Fe (golden), Mn (purple), V (silvery), Ce (yellow).

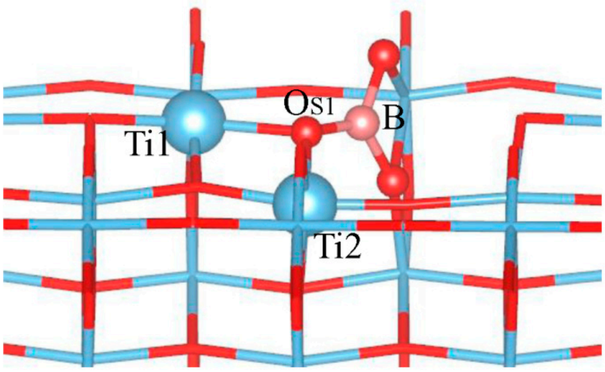

Figure S11. Local structure of anatase (101) surface with the Ti_{5c} site being doped by B, where the Ti-O_{S1} and Ti-O_{S2} bond distances are calculated to be approximately 2.066 and 2.099 Å instead of 1.985 and 2.056 Å at pristine surface.
Color scheme: Ti (blue), O (red), B (pink).

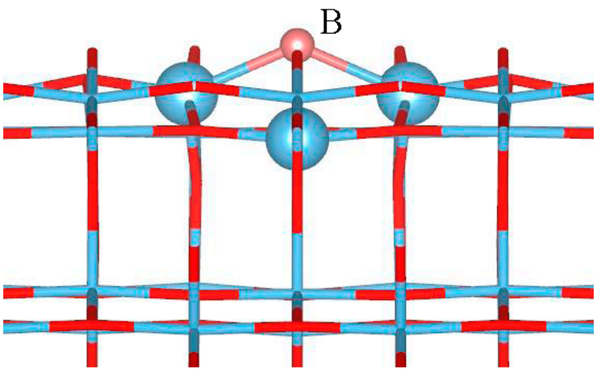

Figure S12. Local structure of anatase (101) surface with the O_{3c} site being doped by B. Color scheme: Ti (blue), O (red), B (pink).

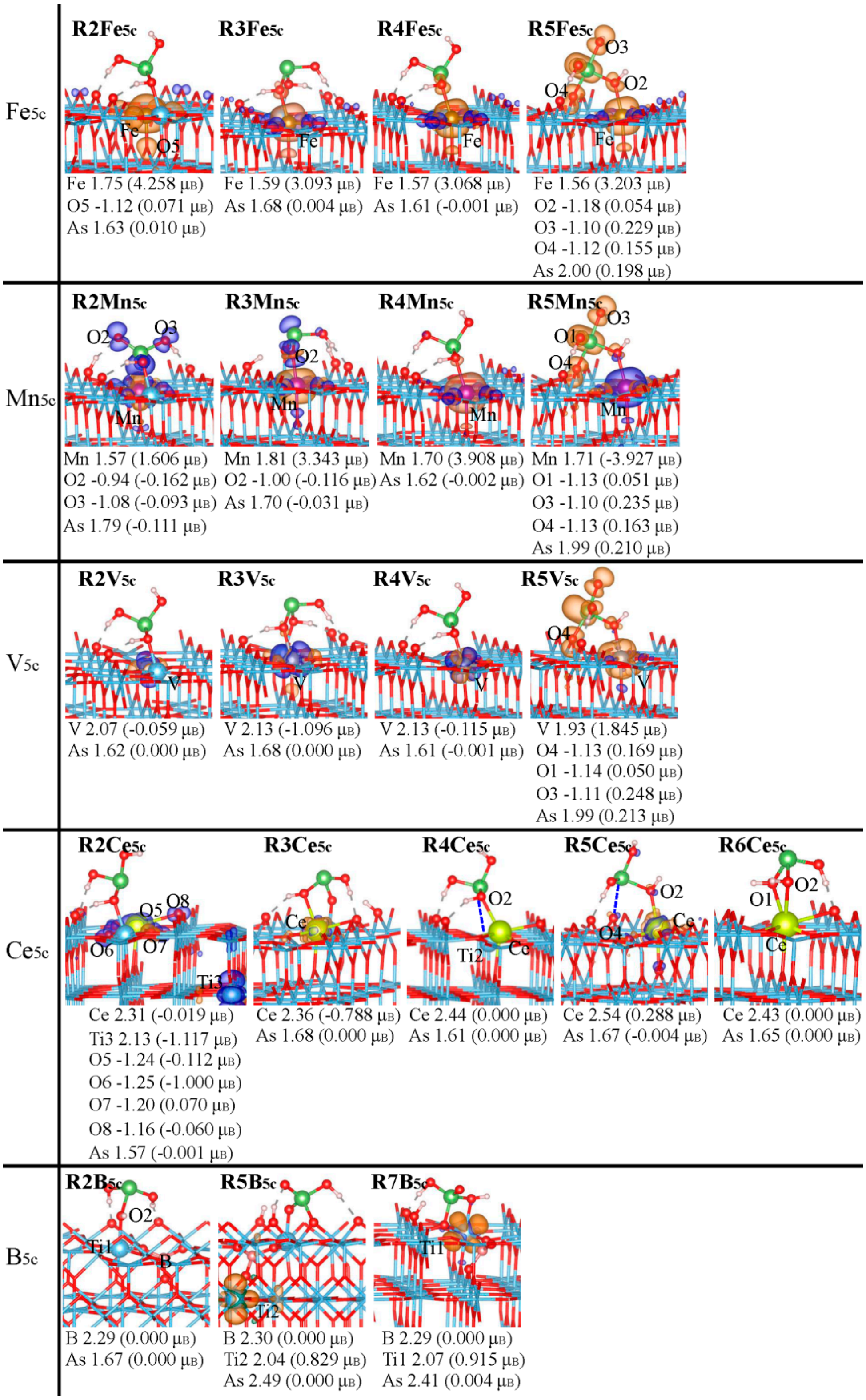

Figure S13. Spin density isosurfaces for $As(OH)_3$ adsorption at rutile (110) surface with the Ti_{5c} site being doped.

Color scheme: Ti (blue), O (red), H (white), Fe (golden), Mn (purple), V (silvery), Ce (yellow), B (pink). "+" and "-" values are visualized in orange and blue, respectively (±0.003 e/Å^3). Bader charges (|e|) and magnetic moments (μ_B, in parentheses) for specific atoms are listed.

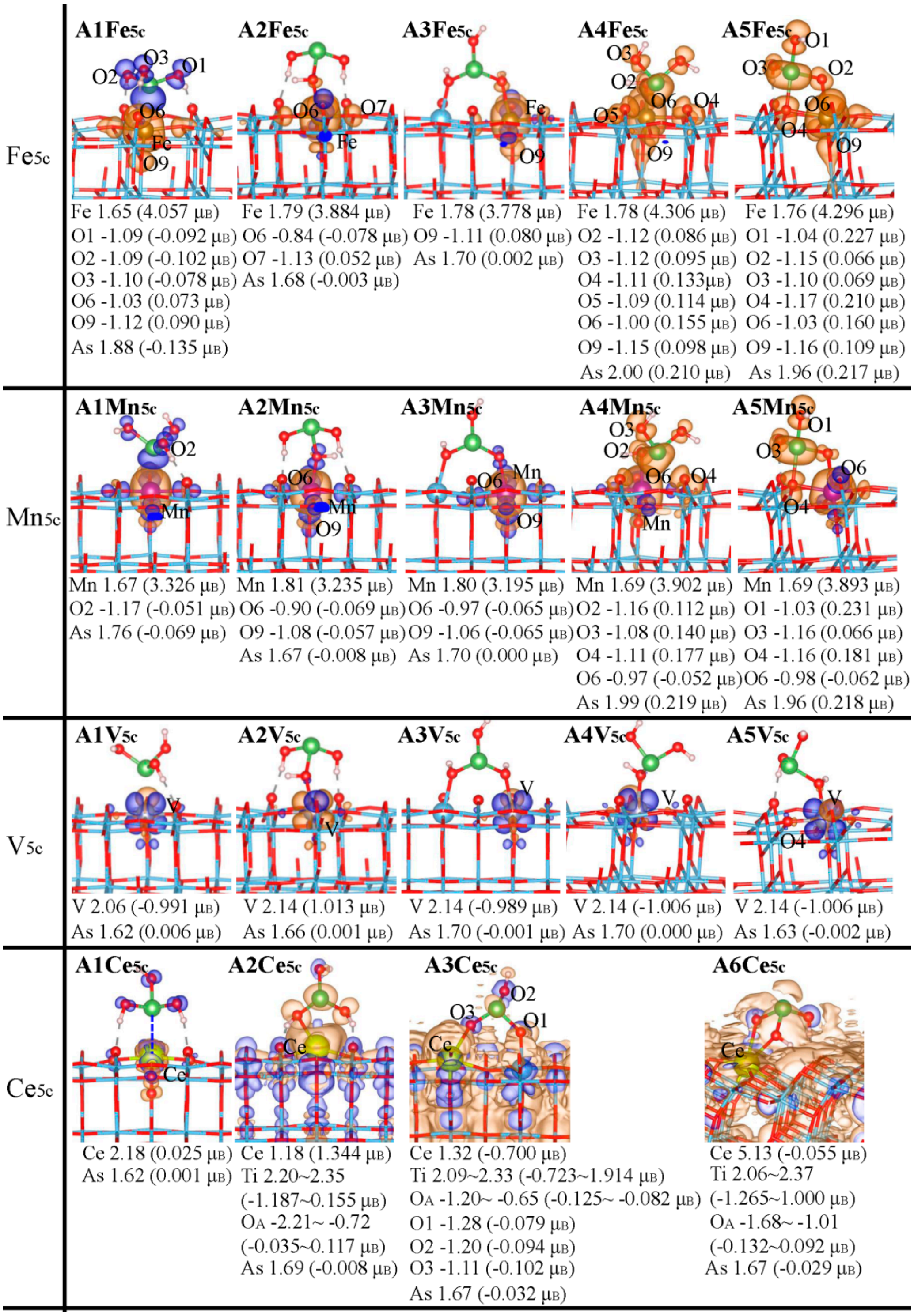
Fe5c
A1Fe5c
O3
O2
O1
O6
Fe
O9
Fe 1.65 (4.057 μB)
O1 -1.09 (-0.092 μB)
O2 -1.09 (-0.102 μB)
O3 -1.10 (-0.078 μB)
O6 -1.03 (0.073 μB)
O9 -1.12 (0.090 μB)
As 1.88 (-0.135 μB)
A2Fe5c
O6
O7
Fe
Fe 1.79 (3.884 μB)
O6 -0.84 (-0.078 μB)
O7 -1.13 (0.052 μB)
As 1.68 (-0.003 μB)
A3Fe5c
Fe
O9
Fe 1.78 (3.778 μB)
O9 -1.11 (0.080 μB)
As 1.70 (0.002 μB)
A4Fe5c
O3
O2
O6
O4
O5
O9
Fe 1.78 (4.306 μB)
O2 -1.12 (0.086 μB)
O3 -1.12 (0.095 μB)
O4 -1.11 (0.133μB)
O5 -1.09 (0.114 μB)
O6 -1.00 (0.155 μB)
O9 -1.15 (0.098 μB)
As 2.00 (0.210 μB)
A5Fe5c
O1
O3
O2
O6
O4
O9
Fe 1.76 (4.296 μB)
O1 -1.04 (0.227 μB)
O2 -1.15 (0.066 μB)
O3 -1.10 (0.069 μB)
O4 -1.17 (0.210 μB)
O6 -1.03 (0.160 μB)
O9 -1.16 (0.109 μB)
As 1.96 (0.217 μB)
Mn5c
A1Mn5c
O2
Mn
Mn 1.67 (3.326 μB)
O2 -1.17 (-0.051 μB)
As 1.76 (-0.069 μB)
A2Mn5c
O6
Mn
O9
Mn 1.81 (3.235 μB)
O6 -0.90 (-0.069 μB)
O9 -1.08 (-0.057 μB)
As 1.67 (-0.008 μB)
A3Mn5c
O6
Mn
O9
Mn 1.80 (3.195 μB)
O6 -0.97 (-0.065 μB)
O9 -1.06 (-0.065 μB)
As 1.70 (0.000 μB)
A4Mn5c
O3
O2
O6
O4
Mn
Mn 1.69 (3.902 μB)
O2 -1.16 (0.112 μB)
O3 -1.08 (0.140 μB)
O4 -1.11 (0.177 μB)
O6 -0.97 (-0.052 μB)
As 1.99 (0.219 μB)
A5Mn5c
O1
O3
O6
O4
Mn 1.69 (3.893 μB)
O1 -1.03 (0.231 μB)
O3 -1.16 (0.066 μB)
O4 -1.16 (0.181 μB)
O6 -0.98 (-0.062 μB)
As 1.96 (0.218 μB)
V5c
A1V5c
V
V 2.06 (-0.991 μB)
As 1.62 (0.006 μB)
A2V5c
V
V 2.14 (1.013 μB)
As 1.66 (0.001 μB)
A3V5c
V
V 2.14 (-0.989 μB)
As 1.70 (-0.001 μB)
A4V5c
V
V 2.14 (-1.006 μB)
As 1.70 (0.000 μB)
A5V5c
V
O4
V 2.14 (-1.006 μB)
As 1.63 (-0.002 μB)
Ce5c
A1Ce5c
Ce
Ce 2.18 (0.025 μB)
As 1.62 (0.001 μB)
A2Ce5c
Ce
Ce 1.18 (1.344 μB)
Ti 2.20~2.35
(-1.187~0.155 μB)
OA -2.21~ -0.72
(-0.035~0.117 μB)
As 1.69 (-0.008 μB)
A3Ce5c
O2
O3
O1
Ce
Ce 1.32 (-0.700 μB)
Ti 2.09~2.33 (-0.723~1.914 μB)
OA -1.20~ -0.65 (-0.125~ -0.082 μB)
O1 -1.28 (-0.079 μB)
O2 -1.20 (-0.094 μB)
O3 -1.11 (-0.102 μB)
As 1.67 (-0.032 μB)
A6Ce5c
Ce
Ce 5.13 (-0.055 μB)
Ti 2.06~2.37
(-1.265~1.000 μB)
OA -1.68~ -1.01
(-0.132~0.092 μB)
As 1.67 (-0.029 μB)

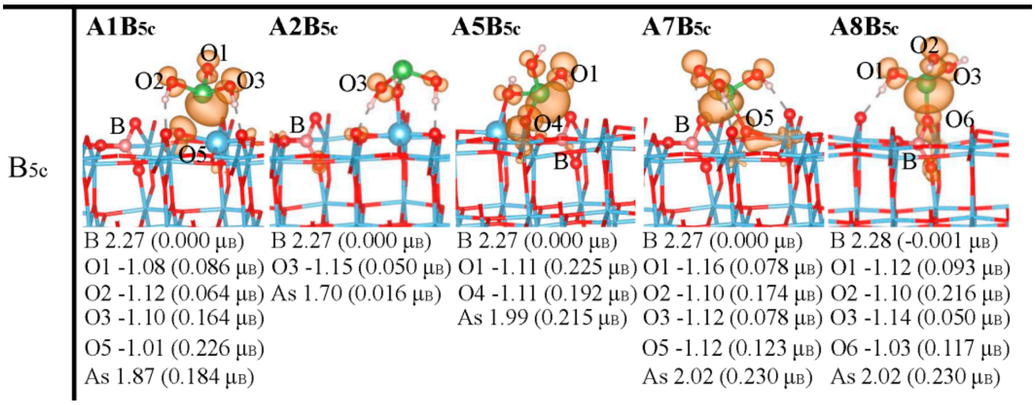

Figure S14. Spin density isosurfaces for $As(OH)_3$ adsorption at anatase (101) surface with the Ti_{5c} site being doped.

Color scheme: Ti (blue), O (red), H (white), Fe (golden), Mn (purple), V (silvery), Ce (yellow), B (pink). For Ce doping, a number of O atoms may be distributed with spin densities and are referred to as OA. "+" and "-" values are visualized in orange and blue, respectively (±0.003 e/Å^3). Bader charges (|e|) and magnetic moments (μ_B, in parentheses) for specific atoms are listed.

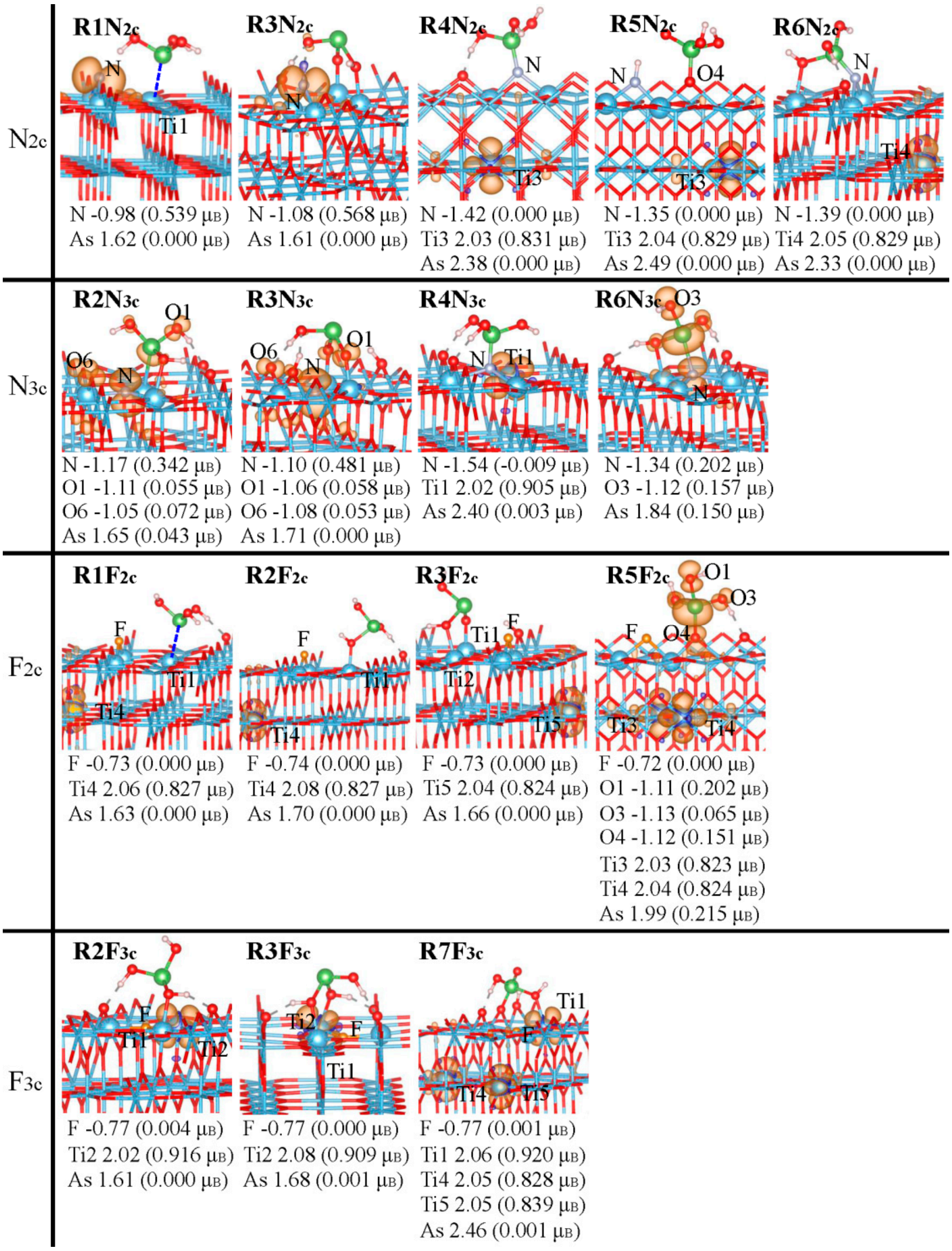
N2c
R1N2c
N
Ti1
N -0.98 (0.539 μB)
As 1.62 (0.000 μB)
R3N2c
N
N -1.08 (0.568 μB)
As 1.61 (0.000 μB)
R4N2c
N
Ti3
N -1.42 (0.000 μB)
Ti3 2.03 (0.831 μB)
As 2.38 (0.000 μB)
R5N2c
N
O4
Ti3
N -1.35 (0.000 μB)
Ti3 2.04 (0.829 μB)
As 2.49 (0.000 μB)
R6N2c
N
Ti4
N -1.39 (0.000 μB)
Ti4 2.05 (0.829 μB)
As 2.33 (0.000 μB)
N3c
R2N3c
O1
O6
N
N -1.17 (0.342 μB)
O1 -1.11 (0.055 μB)
O6 -1.05 (0.072 μB)
As 1.65 (0.043 μB)
R3N3c
O6
O1
N
N -1.10 (0.481 μB)
O1 -1.06 (0.058 μB)
O6 -1.08 (0.053 μB)
As 1.71 (0.000 μB)
R4N3c
N
Ti1
N -1.54 (-0.009 μB)
Ti1 2.02 (0.905 μB)
As 2.40 (0.003 μB)
R6N3c
O3
N
N -1.34 (0.202 μB)
O3 -1.12 (0.157 μB)
As 1.84 (0.150 μB)
F2c
R1F2c
F
Ti1
Ti4
F -0.73 (0.000 μB)
Ti4 2.06 (0.827 μB)
As 1.63 (0.000 μB)
R2F2c
F
Ti1
Ti4
F -0.74 (0.000 μB)
Ti4 2.08 (0.827 μB)
As 1.70 (0.000 μB)
R3F2c
F
Ti1
Ti2
Ti5
F -0.73 (0.000 μB)
Ti5 2.04 (0.824 μB)
As 1.66 (0.000 μB)
R5F2c
O1
O3
F
O4
Ti3
Ti4
F -0.72 (0.000 μB)
O1 -1.11 (0.202 μB)
O3 -1.13 (0.065 μB)
O4 -1.12 (0.151 μB)
Ti3 2.03 (0.823 μB)
Ti4 2.04 (0.824 μB)
As 1.99 (0.215 μB)
F3c
R2F3c
F
Ti1
Ti2
F -0.77 (0.004 μB)
Ti2 2.02 (0.916 μB)
As 1.61 (0.000 μB)
R3F3c
Ti2
F
Ti1
F -0.77 (0.000 μB)
Ti2 2.08 (0.909 μB)
As 1.68 (0.001 μB)
R7F3c
Ti1
F
Ti4
Ti5
F -0.77 (0.001 μB)
Ti1 2.06 (0.920 μB)
Ti4 2.05 (0.828 μB)
Ti5 2.05 (0.839 μB)
As 2.46 (0.001 μB)

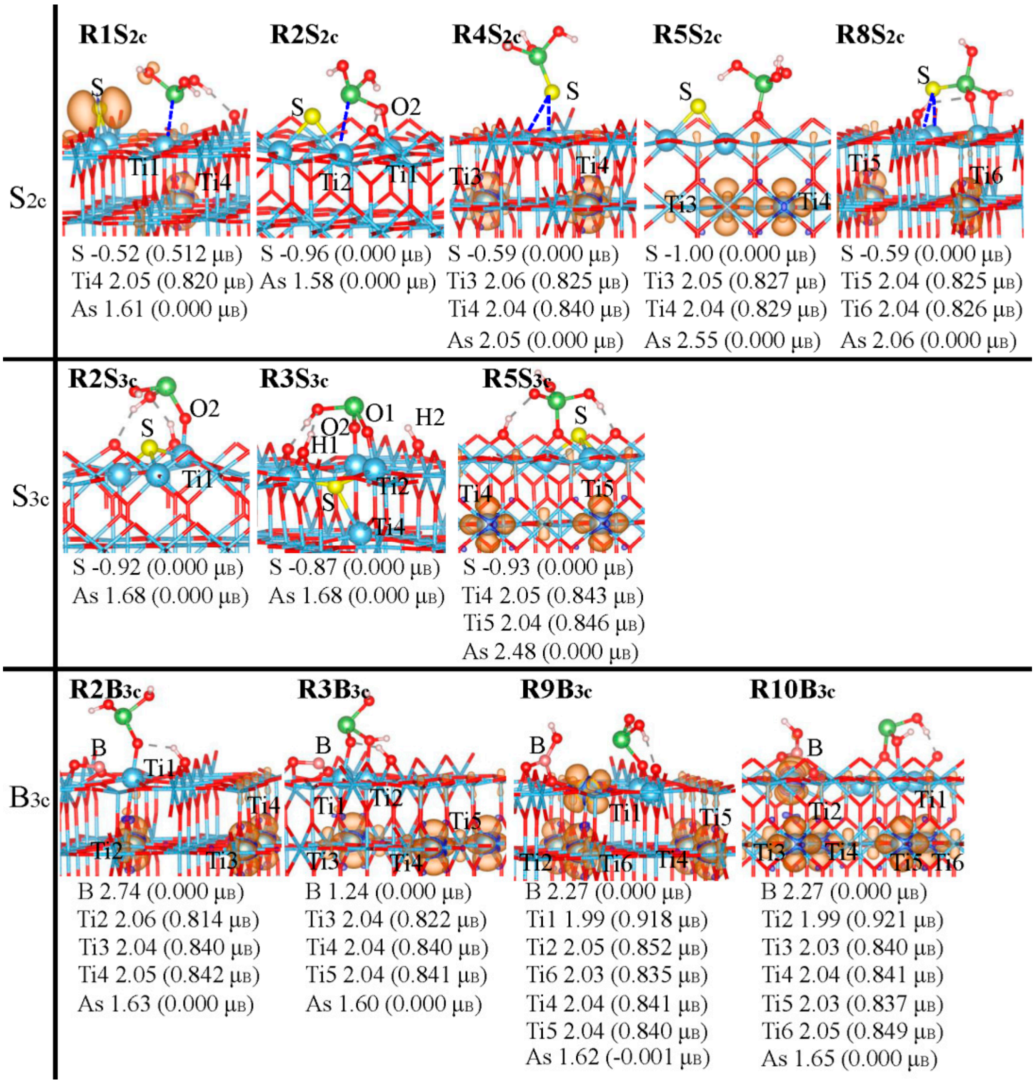

Figure S15. Spin density isosurfaces for $As(OH)_3$ adsorption at rutile (110) surface with the O_{2c}, O_{3c} site being doped.

Color scheme: Ti (blue), O (red), As (green), H (white), N (gray), F (orange), S (yellow), B (pink). "+" and "-" values are visualized in orange and blue, respectively (±0.003 e/Å^3). Bader charges (|e|) and magnetic moments (μ_B, in parentheses) for specific atoms are listed.

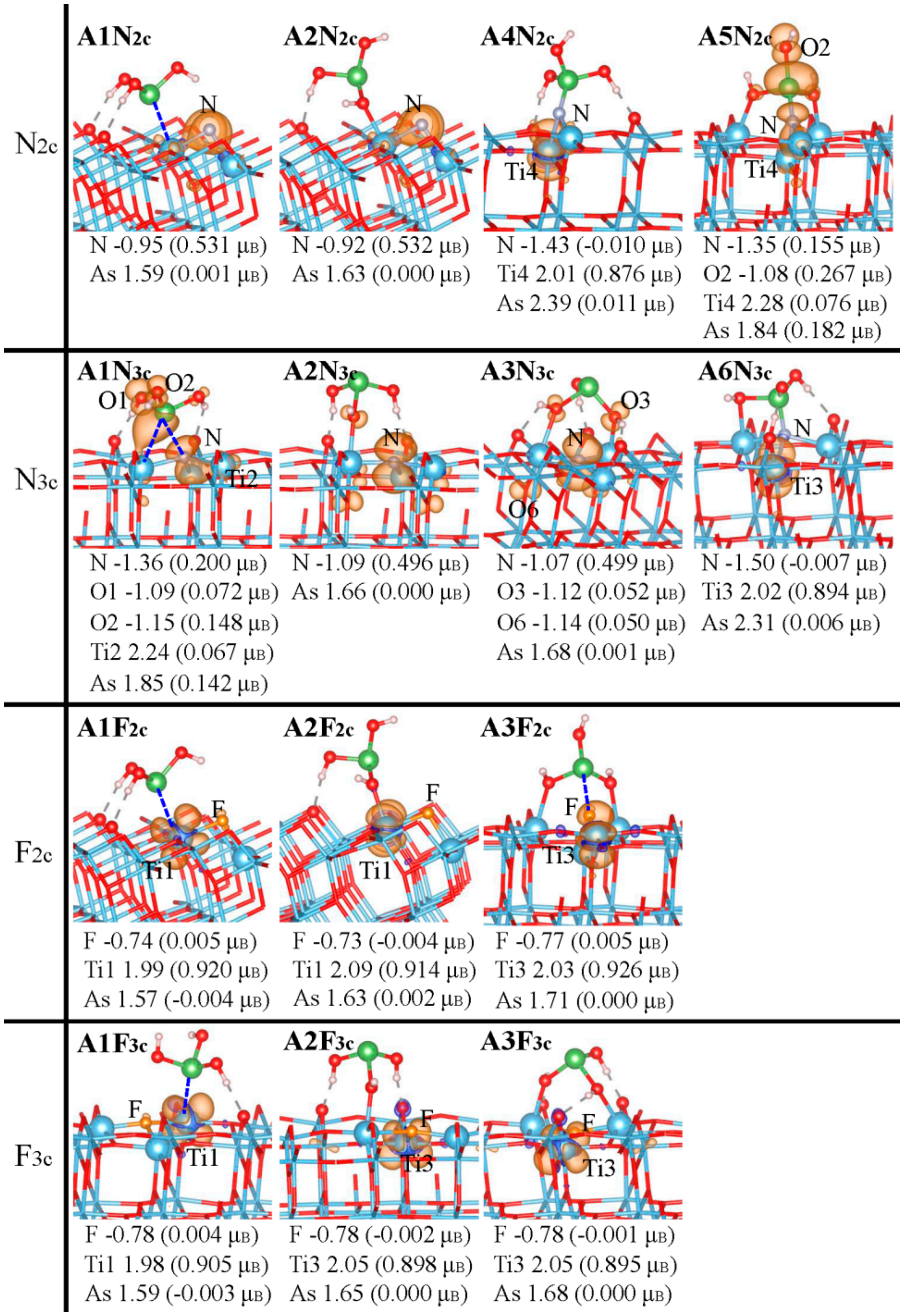
A1N2c
A2N2c
A4N2c
A5N2c
N2c
N
O2
Ti4
N -0.95 (0.531 μB)
As 1.59 (0.001 μB)
N -0.92 (0.532 μB)
As 1.63 (0.000 μB)
N -1.43 (-0.010 μB)
Ti4 2.01 (0.876 μB)
As 2.39 (0.011 μB)
N -1.35 (0.155 μB)
O2 -1.08 (0.267 μB)
Ti4 2.28 (0.076 μB)
As 1.84 (0.182 μB)
A1N3c
A2N3c
A3N3c
A6N3c
N3c
O1
O2
Ti2
O3
O6
Ti3
N -1.36 (0.200 μB)
O1 -1.09 (0.072 μB)
O2 -1.15 (0.148 μB)
Ti2 2.24 (0.067 μB)
As 1.85 (0.142 μB)
N -1.09 (0.496 μB)
As 1.66 (0.000 μB)
N -1.07 (0.499 μB)
O3 -1.12 (0.052 μB)
O6 -1.14 (0.050 μB)
As 1.68 (0.001 μB)
N -1.50 (-0.007 μB)
Ti3 2.02 (0.894 μB)
As 2.31 (0.006 μB)
A1F2c
A2F2c
A3F2c
F2c
F
Ti1
Ti3
F -0.74 (0.005 μB)
Ti1 1.99 (0.920 μB)
As 1.57 (-0.004 μB)
F -0.73 (-0.004 μB)
Ti1 2.09 (0.914 μB)
As 1.63 (0.002 μB)
F -0.77 (0.005 μB)
Ti3 2.03 (0.926 μB)
As 1.71 (0.000 μB)
A1F3c
A2F3c
A3F3c
F3c
F -0.78 (0.004 μB)
Ti1 1.98 (0.905 μB)
As 1.59 (-0.003 μB)
F -0.78 (-0.002 μB)
Ti3 2.05 (0.898 μB)
As 1.65 (0.000 μB)
F -0.78 (-0.001 μB)
Ti3 2.05 (0.895 μB)
As 1.68 (0.000 μB)

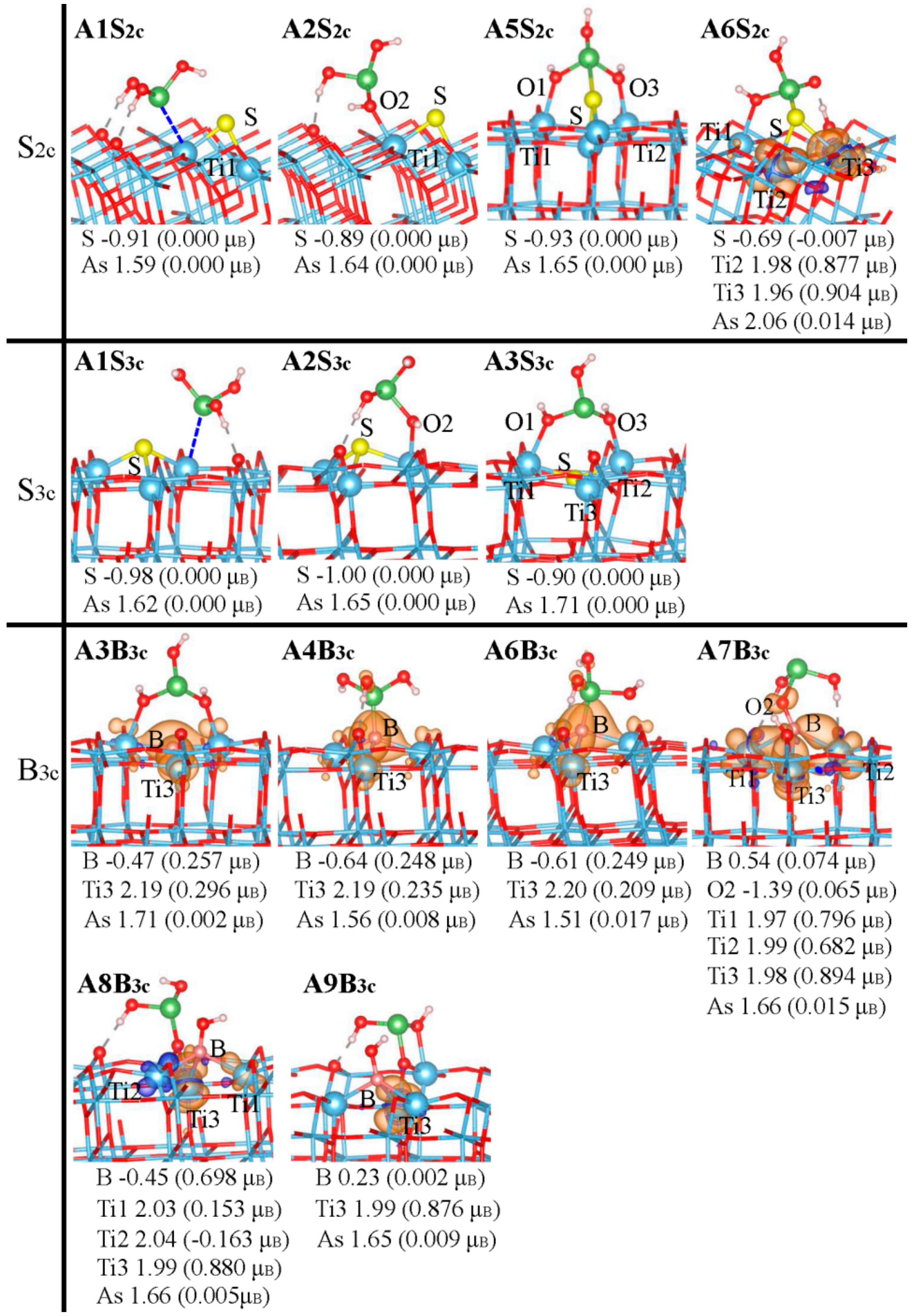

Figure S16. Spin density isosurfaces for $As(OH)_3$ adsorption at anatase (101) surface with the O_{2c} and O_{3c} sites being doped.

Color scheme: Ti (blue), O (red), As (green), H (white), N (gray), F (orange), S (yellow), B (pink). "+" and "-" values are visualized in orange and blue, respectively (±0.003 e/Å^3). Bader charges (|e|) and magnetic moments (μ_B, in parentheses) for specific atoms are listed.

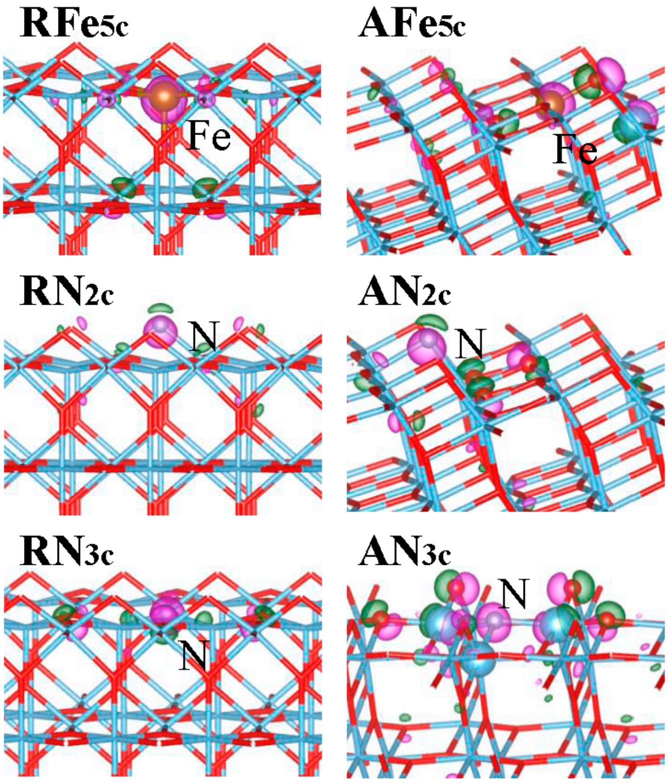

Figure S17. Isosurfaces of charge density difference between doped and pristine forms of rutile (110) and anatase (101) surfaces (D_{Ti} = Fe; D_O = N).

Color scheme: Ti (blue), O (red), N (gray), Fe (golden). "+" and "-" values are visualized in green and purple, respectively (±0.05 e/Å^3).

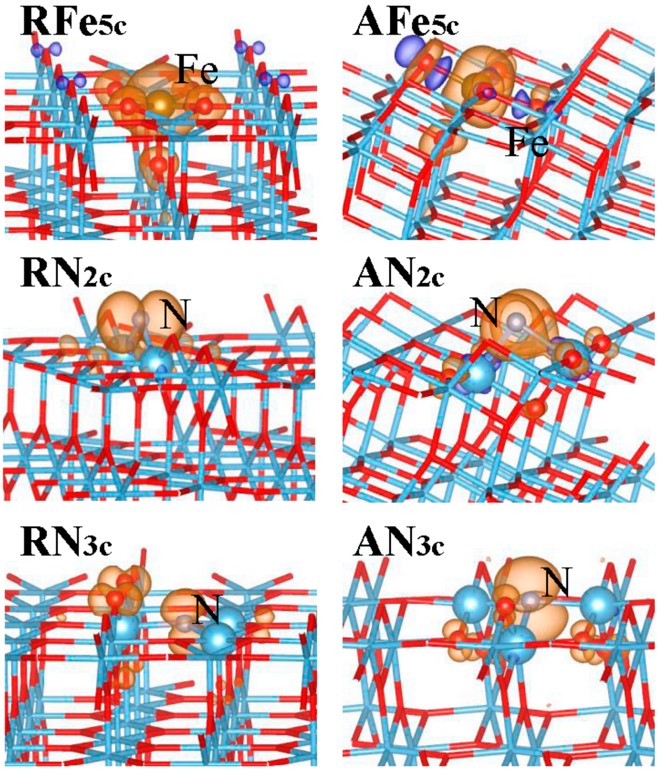

Figure S18. Spin density isosurfaces for rutile (110) and anatase (101) surfaces with the Ti_{5c}, O_{2c} or O_{3c} site being doped (D_{Ti} = Fe; D_O = N).
Color scheme: Ti (blue), O (red), N (gray), Fe (golden). "+" and "-" values are visualized in orange and blue, respectively (±0.003 e/Å^3).

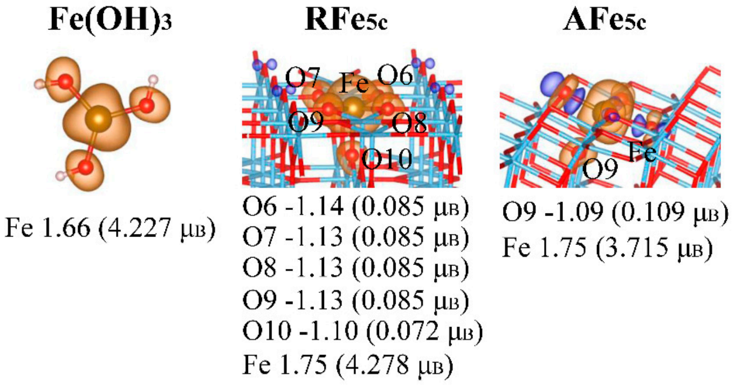

Figure S19. Spin density isosurfaces for $Fe(OH)_3$, and rutile (110) and anatase (101) surfaces with Fe doping at the Ti_{5c} site, as well as Bader charges (|e|) magnetic moments (μ_B, in parentheses) for Fe and proximal O atoms.

Color scheme: Ti (blue), O (red), N (gray), Fe (golden). $Fe(OH)_3$ is used as reference for Fe in +III state. "+" and "-" values are visualized in orange and blue, respectively (±0.003 e/Å^3).

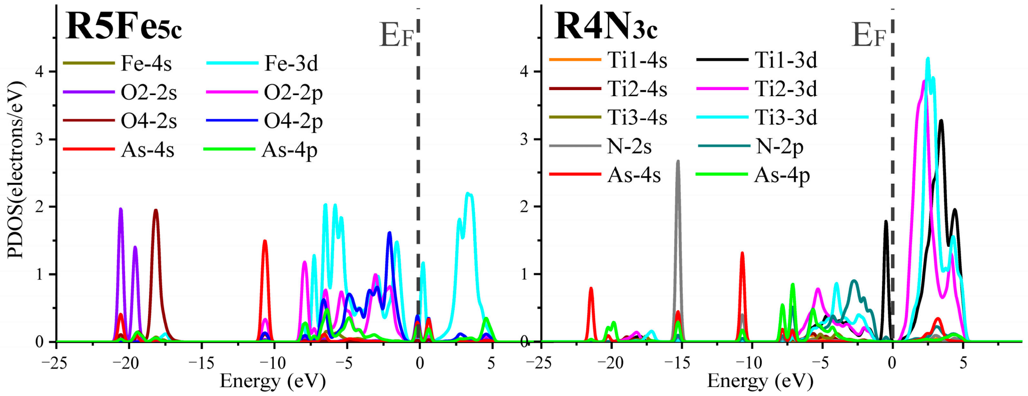

Figure S20. Projected density of states (PDOS) for adsorption configurations of $As(OH)_3$ at rutile (110) with doping the Ti_{5c} (**R5Fe5c**), O_{3c} (**R4N3c**) site.

The Fermi level (E_F) is set to zero energy and highlighted by gray dotted line.
